# Recent Warming, Rather than Industrial Emissions of Bioavailable Nutrients, Is the Dominant Driver of Lake Primary Production Shifts across the Athabasca Oil Sands Region

**DOI:** 10.1371/journal.pone.0153987

**Published:** 2016-05-02

**Authors:** Jamie C. Summers, Joshua Kurek, Jane L. Kirk, Derek C. G. Muir, Xiaowa Wang, Johan A. Wiklund, Colin A. Cooke, Marlene S. Evans, John P. Smol

**Affiliations:** 1 Paleoecological Environmental Assessment and Research Laboratory, Department of Biology, Queen’s University, Kingston, Ontario, Canada; 2 Department of Geography and Environment, Mount Allison University, Sackville, New Brunswick, Canada; 3 Aquatic Contaminants Research Division, Environment Canada, Burlington, Ontario, Canada; 4 Alberta Environmental Monitoring, Evaluation and Reporting Agency, Edmonton, Alberta, Canada; 5 Department of Earth and Atmospheric Sciences, University of Alberta, Edmonton, Alberta, Canada; 6 Water Hydrology Ecology Research Division, Environment Canada, Saskatoon, Saskatchewan, Canada; Institute of Tibetan Plateau Research, CHINA

## Abstract

Freshwaters in the Athabasca Oil Sands Region (AOSR) are vulnerable to the atmospheric emissions and land disturbances caused by the local oil sands industry; however, they are also affected by climate change. Recent observations of increases in aquatic primary production near the main development area have prompted questions about the principal drivers of these limnological changes. Is the enhanced primary production due to deposition of nutrients (nitrogen and phosphorus) from local industry or from recent climatic changes? Here, we use downcore, spectrally-inferred chlorophyll-*a* (VRS-chl*a*) profiles (including diagenetic products) from 23 limnologically-diverse lakes with undisturbed catchments to characterize the pattern of primary production increases in the AOSR. Our aim is to better understand the relative roles of the local oil sands industry versus climate change in driving aquatic primary production trends. Nutrient deposition maps, generated using geostatistical interpolations of spring-time snowpack measurements from a grid pattern across the AOSR, demonstrate patterns of elevated total phosphorus, total nitrogen, and bioavailable nitrogen deposition around the main area of industrial activity. However, this pattern is not observed for bioavailable phosphorus. Our paleolimnological findings demonstrate consistently greater VRS-chl*a* concentrations compared to pre-oil sands development levels, regardless of morphological and limnological characteristics, landscape position, bioavailable nutrient deposition, and dibenzothiophene (DBT)-inferred industrial impacts. Furthermore, breakpoint analyses on VRS-chl*a* concentrations across a gradient of DBT-inferred industrial impact show limited evidence of a contemporaneous change among lakes. Despite the contribution of bioavailable nitrogen to the landscape from industrial activities, we find no consistency in the spatial pattern and timing of VRS-chl*a* shifts with an industrial fertilizing signal. Instead, significant positive correlations were observed between VRS-chl*a* and annual and seasonal temperatures. Our findings suggest warmer air temperatures and likely decreased ice covers are important drivers of enhanced aquatic primary production across the AOSR.

## Introduction

Deposits of bitumen in northeastern Alberta are immense. Estimates indicate ~167 billion barrels of oil underlie ~140,000 km^2^ of boreal forest [1]. Of Alberta’s three major bitumen deposits, the Athabasca deposit is the largest, accounting for ~66% of Alberta’s total oil sands area and ~80% of current oil production [2,3]. The Athabasca deposit hosts the only significant Canadian bitumen deposit accessible via surface mining as well as *in situ* techniques.

Since the late-1960s, the development of Alberta’s oil sands industry has expanded rapidly [4]. As continued industrial growth is forecasted [5,6], freshwaters in the Athabasca Oil Sands Region (AOSR) are vulnerable to pollutant emissions, wind-blown dust, land disturbance, and declines in water quality and quantity associated with local industrial activities [7,8]. However, regional freshwaters are also sensitive to the shifts in air temperature (~1.65°C increase in average air temperature since 1960) and precipitation patterns (increased evaporation and glacial melt, and decreased snowpack and river flows) that have occurred in the region during the 20^th^ century [9]. It is expected that climate change will have substantial effects on the ecology and biological production of the shallow lakes in the western Boreal Plain through both abiotic and biotic controls [10,11].

Paleolimnological studies can effectively compare conditions before and after disturbances, such as industrial activity, and are thus useful in the absence of and in combination with monitoring data [12]. Paleolimnological studies have played an integral role in assessing recent environmental changes in the AOSR. For example, studies have noted that aquatic systems in the AOSR and those nearby have shifted in ecological structure and function [13–17]. A recent study used dated lake sediment cores to demonstrate that sedimentary chlorophyll *a* and its diagenetic products (hereafter chl*a*), a proxy for whole lake primary production, have substantially increased since the 1970s in five shallow lakes located up to ~50 km away from the main development area of the AOSR [15]. Shifts in lake structure and function were also reflected by higher trophic levels as algal-grazing zooplankton such as *Daphnia* increased in both occurrence and relative abundance, despite increased contaminant deposition [15]. Changes in diatom assemblage composition [13], diatom valve flux [17], stable carbon (δ^13^C) and nitrogen (δ^15^N) isotopes, and nutrient ratios (C:N) [14] in lakes across the broader region (up to ~350 km from the main development area) also support the findings of enhanced primary production in many of these systems during recent decades.

While many lakes in the AOSR are currently moderately to highly productive [13,15], increases in primary production may reflect the impacts from expanding industrialization. Bioavailable sources of reactive nitrogen from industrial upgrader emissions and wind-blown dust rich in cations and phosphorus from disturbed landscapes could also stimulate lake primary production [18,19]. Analyses of snowpack [20–22], lichen [23], moss [24], and soil [19] from the AOSR identified atmospheric deposition of metals and other pollutants from industrial oil sands activities. Kirk *et al*. [22] estimated 28.6 tonnes of total phosphorus and 463 tonnes of total nitrogen, among other particulates, were deposited in the spring snowpack (winter 2011–2012) within a ~50-km radius of the main industrial upgrading facilities, suggesting that increased loading of bioavailable nutrients may also be occurring across the region.

Alternatively, increases in primary production in shallow lakes of the AOSR may reflect a regional stressor, such as climate change, as the key driver of lake shifts [15]. Climate directly affects the physical properties of lakes and indirectly influences the availability of key resources for primary production, including light, nutrients, and habitat [25,26]. Temperature-mediated processes can affect primary production and subsequent concentrations of chl*a* in aquatic ecosystems. Increasing temperatures can, for example, reduce ice cover and lengthen the growing season, altering light availability, nutrient dynamics, and turbulence conditions [27,28]. Warmer temperatures can also alter the evaporation-to-precipitation ratio, and concentrate nutrients as well as ions and pollutants, with shallow lakes being particularly sensitive given their high surface area-to-volume ratio [29]. Precipitation-mediated processes affect transport of allochthonous materials into lakes, and influence water levels, water colour, and nutrient availability [11,30,31]. Reduced water levels can concentrate nutrients [32] and, depending on turbidity, promote light penetration to greater depths, thereby facilitating an expanded zone of aquatic primary production [33]. Additionally, windy conditions may promote resuspension of nutrients and subsequent growth of phytoplankton, although the effect is dependent on the size and depth of a lake [34,35].

Shallow lakes and wetlands comprise a large portion of the AOSR landscape. Although alternate equilibria in shallow lakes can dampen or delay ecological responses, the relatively small volumes and lower dilution potential in shallow systems can predictably accelerate and/or amplify their response to environmental stressors such as contaminant deposition and climate change [36]. Increases in primary production in AOSR lakes could thus indicate water quality shifts across the region.

**Fig 1:**
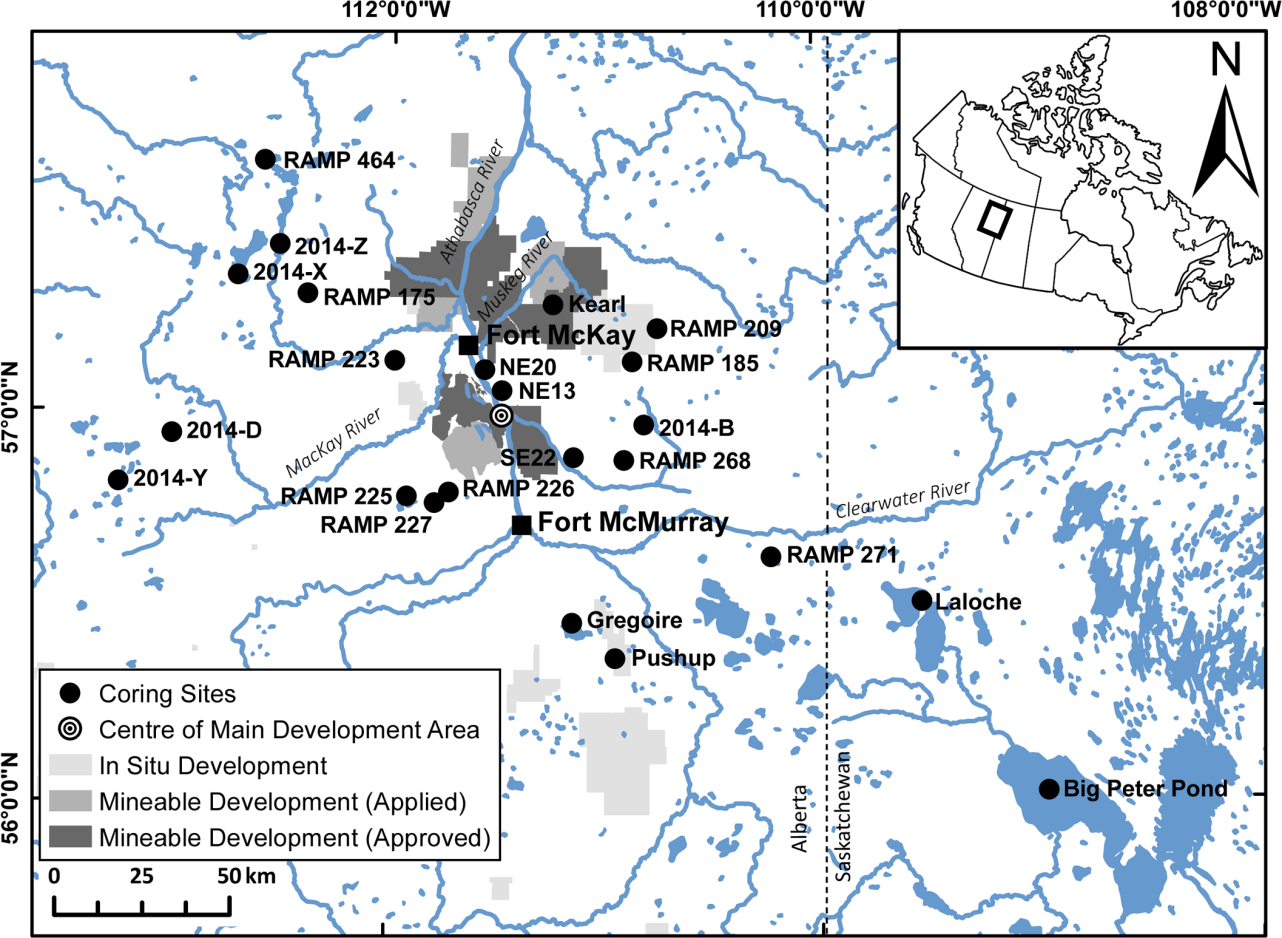
Map of study area. Locations of 23 study lakes and two local communities (Fort McMurray and Fort McKay), and the footprint of industrial oil sands development. Lakes were cored in March 2011, 2012, 2013, or 2014. Many of the lakes have multiple names (S1 Table).

This study uses dated cores from 23 limnologically-diverse lakes (S1 and S2 Tables) in undisturbed catchments located ~10 to 200 km away from the main development area (Fig 1) to characterize spatial patterns in primary production across the AOSR. Maps of nutrient deposition across the AOSR, developed from snowpack samples, are used to compare spatial patterns of nutrient deposition with the changes in aquatic primary production. We reason that an industrial fertilizing effect will diminish with declining aerial deposition and increased distance from industrial oil sands developments, while the effect of a climatic driver will persist across the region. With forecasts of continued climatic changes and intensifying industrial development in the AOSR, it is plausible that further ecosystem shifts will occur. Understanding the long-term dynamics and key drivers of this primary production increase is crucial in shaping the management strategies of freshwater resources in the AOSR.

## Materials and Methods

### Sample collection and ^210^Pb dating

Sample collection and dating methods follow the procedure reported in Kurek *et al*. [15]. Sediment cores from 23 limnologically-diverse lakes (S2 Table) within a ~200-km radius of the main development area in the AOSR were collected through augered ice holes at the deepest part of the lakes in March 2011, 2012, 2013, and 2014. Samples were collected as part of the Canada-Alberta Joint Oil Sands Monitoring (JOSM) program (http://jointoilsandsmonitoring.ca) and were taken from both phosphorus and nitrogen-limited lakes (S2 Table) with undisturbed catchments. Lakes were accessed by helicopter and were located on provincially-owned, publically-accessible lands. No private lands or reserve areas were accessed, no protected species were sampled, and no permits or permissions were required.

Cores were collected using a Uwitec gravity corer, specially designed for high-resolution work on recent sediments, and the sediment profiles were sectioned at 0.5-cm intervals for the upper 20 cm, and 1-cm intervals thereafter. Sediment intervals were sampled into Nalgene jars or Whirlpak^®^ bags and immediately frozen. Determination of total ^210^Pb, ^226^Ra and ^137^Cs activity was completed by Flett Research Ltd. (Winnipeg, Manitoba, Canada). ^210^Po and ^226^Ra alpha radiation was measured as a proxy for total ^210^Pb and supported^210^Pb activity respectively [37]. ^137^Cs activity was measured via gamma spectrometry and was used as an independent chronological marker of the radioactive fallout peak following the 1963 moratorium on nuclear weapons testing [37]. The constant rate of supply (CRS) age model [37] was used to estimate sediment core age-depth relations (± 2 SD) for all analyzed intervals (where total ^210^Pb > supported ^210^Pb) and the dry mass accumulation rate for each core was used to extrapolate the sediment ages below background depth. Lastly, to assign a date to every interval, a polynomial regression was fitted to the previously-determined dates using the lowest order polynomial that provided a reasonable fit (S1 Fig).

### Chlorophyll *a* inferences using visible reflectance spectroscopy (VRS-chl*a*)

Visible reflectance spectroscopy (VRS) was used to estimate concentrations of sedimentary chl*a*, which is a proxy for whole lake primary production [38,39]. VRS methods measure the entire suite of chl*a* degradation products; therefore, the spectral inferences are not affected by post-depositional diagenetic processes [38,39]. The use of VRS-chl*a* determinations has now been assessed in a variety of limnological settings and has been shown to be a reliable method for estimating trends in whole-lake primary production [39]. For example, it has faithfully tracked recent increases in production with known fertilization events [40], recent declines in primary production as a result of reduced upwelling of hypolimnetic nutrients due to enhanced thermal stability [41], and the effects of past algaecide treatments on production [42].

In preparation for spectral analysis, sediments were freeze-dried, sieved through a 125-μm mesh, and the spectral reflectance of ~2–3 mm of sediment in a 19 x 65 mm glass vial was analyzed in a FOSS NIRSystem Model 6500 rapid content analyzer. A simple reflectance metric (the area under the absorbance peak from 650–700 nm) was used to infer chl*a* concentrations using a linear equation derived from an earlier calibration study [39]. The estimated detection limit of this technique is ~0.01 mg/g dry weight.

### Dibenzothiophenes (DBTs)

Dibenzothiophenes (DBTs) are a group of alkylated polycyclic aromatic compounds (PACs) that are characteristic of AOSR bitumen [43,44]. Studies show DBT deposition in snow in the AOSR declines as a proportion of total PACs with increased distance from industrial development, while unsubstituted PACs show no geographical trend. Thus, DBTs are considered indicative of industrial activities in the region [15,43]. Downcore DBTs were analyzed by AXYS Analytical Services (Sidney, British Columbia, Canada) using a standardized method for PACs (MLA-021) based on the United States Environmental Protection Agency (US EPA) methods 1625B and 8270C/D. Concentrations of DBTs were calculated using the isotope dilution method of quantification. Five DBT analytes (Total DBTs = DBT + C1-C4-alkylated DBTs) were isolated. Additional details on PAC analysis in the sediment cores are given in Kurek *et al*. [15]. A DBT enrichment factor, which is explained below, was used to characterize industrial impact at each site.

### Analysis and mapping of nutrients in snow

To assess aerial inputs of nutrients across the landscape, loadings of total nitrogen (TN), dissolved inorganic nitrogen (DIN), total phosphorus (TP), total dissolved phosphorus (TDP), and soluble reactive phosphorus (SRP) were measured in melted snow samples collected in March 2014. Snow was collected from ~135 sites located in a grid pattern up to 200 km from the major area of development (S2 Fig) using methods described in Kirk *et al*. [22]. TN and TP include both bioavailable and unavailable forms of nitrogen and phosphorus, respectively. DIN includes ammonia, nitrate, and nitrite, and thus represents bioavailable forms. Phytoplankton use orthophosphate, which is measured in SRP, and are also able to assimilate dissolved fractions of phosphorus, which is measured as TDP [45]; therefore, SRP and TDP represent biologically-available forms. All samples were collected within 6 days in early March to ensure maximum snowpack depth and minimize snow variability over the course of sampling. Timing of sampling was selected based on historical snow accumulation data for the Fort McMurray region (Environment Canada, www.ec.gc.ca/dccha-ahccd). Composite snowpack profiles were collected using a pre-cleaned stainless steel corer into pre-cleaned high density polypropylene buckets using clean techniques as in Kirk *et al*. [22]. Analyses were completed at the National Laboratory for Environmental Testing (NLET), which is certified by the Canadian Association for Environmental Analytical Laboratories (CAEAL) using method 200.8 from the US EPA. Ten snow cores were collected at each site to determine average snow-water equivalence (SWE), which was then multiplied by nutrient concentrations at each site to calculate loadings, as described previously in Kirk *et al*. [22] and Kelly *et al*. [20]. DIN snowpack loadings for 1978 were also available for 60 sites (S2 Fig) located varying distances from the main area of industry [46]. Numerous site locations were similar among 1978 and 2014 and methods for sample collection were comparable. ArcGIS10 Geostatistical Analyst software (Esri, Redlands, California) was used to interpolate 2014 spring-time loadings of all nutrients (TN, DIN, TP, TDP, SRP) for an area ~42,800 km^2^, and 1978 DIN spring-time loading for an area ~23,800 km^2^. Kriging settings and comparisons for each interpolated parameter were similar to methods described in Kirk *et al*. [22] and are provided in S3–S5 Tables.

### Numerical analysis

To facilitate comparisons of inferred primary production among lakes, standardized Z scores ((VRS-chl*a* concentration–mean VRS-chl*a* concentration)/standard deviation) were calculated for the VRS-chl*a* concentrations. Enrichment factors were calculated to compare VRS-chl*a* concentrations from the most recent sediment intervals (post-2000; n = 3–12 intervals per core) to concentrations ~15 years prior to industrial development in the region (1955–1970; n = 1–5 intervals per core). The ~15-year pre-industry time period was used because all sites had sediments dating back to at least 1955. To validate the appropriateness of the time intervals selected, enrichment factors were also calculated using the 2 and 3 most-recent intervals and the 2 and 3 intervals immediately preceding 1970. The various methods of calculating the enrichment factors yielded similar results, suggesting that the first method outlined was representative of the changes in VRS-chl*a* concentrations occurring since industrial oil sands development.

In addition to measures of proximity to industry and landscape position, DBT enrichment factors were used to characterize the industrial oil sands impacts at different sites. Although DBT enrichment factors represent the changing sedimentary concentrations of only one industrial contaminant, DBTs are the most representative indication of industrial oil sands activity in the AOSR [15,43]. Enrichment factors of DBTs are calculated from measures of the industrial contaminant in the sediments at each site, and are thus able to acknowledge the AOSR as a region of industry rather than a single facility and incorporate the influence of upwind/downwind landscape position and variable wind conditions on a site’s industrial impact. RAMP 227 was excluded because the DBT data did not extend to pre-industrial times (no measures for 1955–1970).

Sites were then assigned to minimally DBT-enriched (DBT enrichment <2) or highly DBT-enriched groups (DBT enrichment >2). Given that there are no sedimentary guidelines for DBTs [47], we set the point of division for the categories at 2. The point of division was selected based on evidence that DBTs are generated locally [15], but to also account for possible long-range transport of DBTs from outside the AOSR. An independent two-sample *t*-test was used to identify any significant difference between the average VRS-chl*a* enrichments of the highly and minimally DBT-enriched groups.

To characterize the timing of VRS-chl*a* increases, piecewise linear regression models were applied to the VRS-chl*a* concentration data (SigmaPlot Version 10). A linear relationship with a single breakpoint was assumed, and a two-segmented model was used. An ANOVA table and corresponding *F* test statistic from a null model was used to evaluate the statistical significance for each regression model. Breakpoint analyses were not completed on lakes where no stable baseline was captured (RAMP 175, RAMP 226, and 2014-B), where outliers would drive the timing of the breakpoint (Big Peter Pond), and where no DBT enrichment factor could be calculated (RAMP 227).

“Vegan” [48], “analogue” [49], and “Hmisc” [50] R packages [51] were used to calculate Pearson correlations between VRS-chl*a* enrichment factors, DBT-inferred industrial impact, and proximity to industry. Proximity to industry was defined as the distance to snow sampling site AR6, which is adjacent the Athabasca River and two major bitumen upgraders, and has been commonly used to represent the centre of industrial activities within the AOSR (e.g., [20–22]). Further Pearson correlation analyses were carried out on VRS-chl*a* Z scores from minimally and highly DBT-enriched sites versus mean annual and seasonal air temperature (Fort McMurray, station no. 3062696) and precipitation (Fort McMurray, station no. 3062693) data from Environment Canada’s Adjusted and Homogenized Canadian Climate Data website (www.ec.gc.ca/dccha-ahccd). Given downcore sediment compaction and variation in sediment accumulation rates, temporal resolution of samples are reduced down core. To ensure each interval represented comparable amounts of time, the VRS-chl*a* Z scores from cores in each group of DBT enrichment, and climate data were averaged across 5-year intervals (S3 and S4 Figs). Based on the ^210^Pb-estimated ages, most sediment intervals from highly DBT-enriched and minimally DBT-enriched cores represented less than 5 years. Therefore, resolution for VRS-chl*a* Z scores and climate data averages was set to 5 years. Data from intervals representing more than 5 years were retained in the analyses because they represent data across the estimated time frames.

## Results

**Fig 2:**
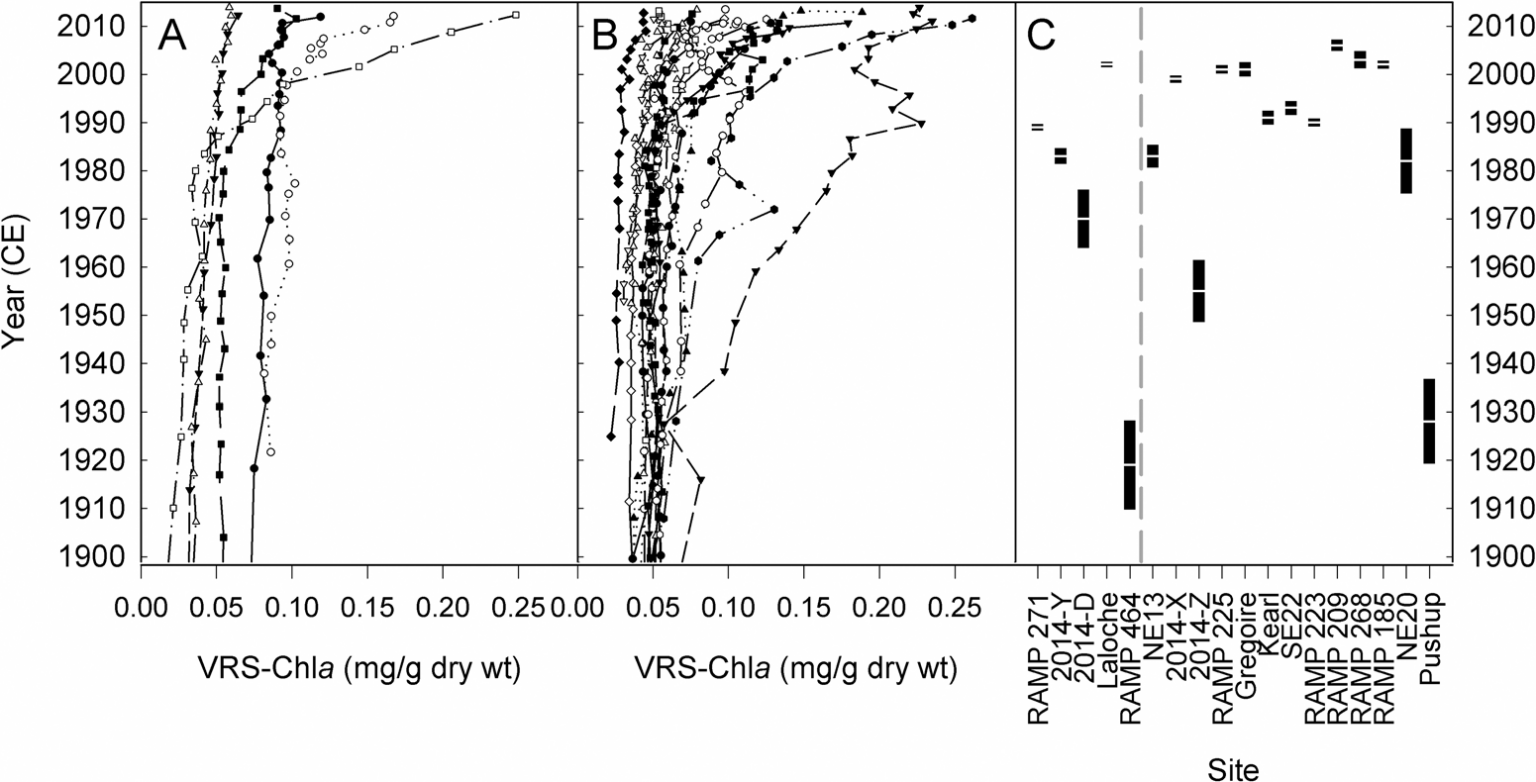
Changes in VRS-chl*a* from each study site. Downcore profiles of VRS-chl*a* concentrations from (A) minimally DBT-enriched sites and (B) highly DBT-enriched sites. Individual profiles are provided in S5 Fig. Timing of abrupt change in VRS-chl*a* concentrations for all sites where breakpoint analysis was applicable, ordered by DBT-enrichment factor are shown in (C). Black rectangles with white midline denote timing of breakpoint and standard error. Sites (n = 5) left of dashed horizontal line are minimally DBT-enriched sites. Sites (n = 13) right of dashed horizontal line are highly DBT-enriched sites.

Modern primary production, inferred from sedimentary chl*a*, is greater than background values at all sites, regardless of proximity to industry (Fig 2, S5 Fig). Most of the 23 sites record relatively stable background (generally pre-1970s) VRS-chl*a* concentrations followed by abrupt increases in surface sediments. Unlike the other sites, RAMP 175 shows a decreasing VRS-chl*a* trend beginning ~2005. Similarly, RAMP 226 shows a decreasing trend from the late-1990s to the late-2000s with a subsequent return to early-1990s concentrations by 2011. Pushup Lake also shows a slight decrease in VRS-chl*a* from the late-1990s to the mid-2000s (S5 Fig). Although VRS-chl*a* profiles from these lakes differ from the regional patterns of consistent primary production increases, modern VRS-chl*a* values are still higher than the pre-oil sands development values at these three sites, resulting in enrichment factors >1. VRS-chl*a* enrichment factors were >1 at all 23 sites, averaging 1.8 (range 1.1 to 5.3) (Table 1).

**Table 1:** Enrichment factors from minimally and highly DBT-enriched sites. VRS-chl*a* enrichment factors, corresponding DBT enrichment factors, and average post-2000 DBT concentrations, organized by DBT enrichment factor. Asterisks (*) denote sites for which breakpoint analysis was not considered appropriate and was thus not applied. RAMP 227 is excluded because the DBT record does not extend to pre-oil sands industry.

Site	Chl*a* Enrichment Factor	DBT Enrichment Factor	Average [DBT] Post 2000	DBT Enrichment Group
			(ng/g dry weight)	
RAMP 271	1.9	0.7	80.4	
2014-Y	1.6	1.1	21.1	Minimally
Big Peter Pond*	2.3	1.5	21.4	DBT-enriched
2014-D	2.1	1.6	29.8	Sites
Laloche	1.4	1.8	34.2	
RAMP 464	1.3	1.9	49.7	
NE13	5.3	2.1	176.4	
RAMP 226*	1.3	2.3	117.9	
2014-X	1.3	2.4	26.5	
2014-Z	1.5	2.6	28.3	
RAMP 225	2.6	2.7	124.3	
Gregoire	1.3	2.7	92.5	
RAMP 175*	2.2	2.9	62.7	
Kearl	1.3	3.1	388.2	Highly
SE22	1.2	3.9	213.2	DBT-enriched
RAMP 223	1.3	5.1	147.9	Sites
RAMP 209	1.3	6.2	162.2	
RAMP 268	1.7	7.1	324.9	
2014-B*	2.0	7.5	290.8	
RAMP 185	1.5	8.6	231.0	
NE20	1.7	22.8	1235.0	
Pushup	1.3	37.4	101.9	

DBT enrichment factors exceeded 1 at all sites except RAMP 271 (0.7), averaging 4.4 (range 0.7 to 37.4) (Table 1). Generally, DBT-enrichment factors are greater east of the main development area and within the 50-km zone of high contaminant deposition identified in the study by Kelly *et al*. [20]; however, highly DBT-enriched lakes are not necessarily closer to the main area of industrial activity (Pearson correlation results for DBT enrichment factor versus distance to the main area of development: r = -0.4, p = 0.06). Pushup Lake demonstrates the largest DBT enrichment factor due to a rapid increase to moderately high DBT concentrations from low pre-oil sands industry levels. The low pre-oil sands industry DBT concentrations at Pushup Lake contribute to the inflated DBT enrichment factor at this relatively distant site. NE20, which is close to the main area of industrial development and demonstrates the second-highest DBT enrichment factor, has modern DBT concentrations over an order of magnitude greater than Pushup Lake.

The average VRS-chl*a* enrichment factors (Table 1) are not significantly different among the minimally and highly DBT-enriched groups (independent two-sample t(5) = 0.35, p = 0.74). Further, no significant correlation was found between chl*a* enrichment factors and proximity to the main area of industrial oil sands development (r = -0.23, p = 0.29) or DBT-inferred industrial impact (r = -0.23, p = 0.31). Breakpoint analyses on VRS-chl*a* concentrations from each lake (except RAMP 175, RAMP 226, 2014-B, Big Peter Pond, and RAMP 227 for reasons described in Materials and Methods) identified abrupt changes ranging from ~1919 to ~2006 (Fig 2, S5 Fig), with 83% (15 out of 18) of the lakes experiencing an abrupt change at ~1970 or later (Fig 2, S5 Fig).

Pearson correlation analyses identified significant (p < 0.05) positive correlations between mean annual and seasonal air temperatures, and VRS-chl*a* Z scores in the highly DBT-enriched group and all sites combined (Table 2). Positive, significant correlations were also identified between mean annual, winter, summer, and fall air temperatures, and VRS-chl*a* Z scores in the minimally DBT-enriched group. No significant correlations were identified between VRS-chl*a* Z scores and mean annual and seasonal precipitation (S6 Table).

**Table 2:** Correlations between 5-year averaged VRS-chl*a* Z scores and 5-year averaged temperature data. Results from Pearson correlations between VRS-chl*a* Z scores (averaged over 5-year intervals) from highly and minimally DBT-enriched sites and all sites combined and annual and seasonal AOSR temperature (averaged over the same 5-year intervals).

		Temperature				
		Annual	Winter	Spring	Summer	Fall
**Highly DBT-Enriched Sites**	**r**	0.71	0.63	0.50	0.72	0.56
	**p**	0.001	0.005	0.03	0.001	0.02
**Minimally DBT- Enriched Sites**	**r**	0.68	0.62	0.44	0.68	0.62
	**P**	0.002	0.01	0.07	0.002	0.01
**All Sites**	**r**	0.70	0.63	0.47	0.70	0.59
	**p**	0.001	0.005	0.047	0.001	0.01

**Fig 3:**
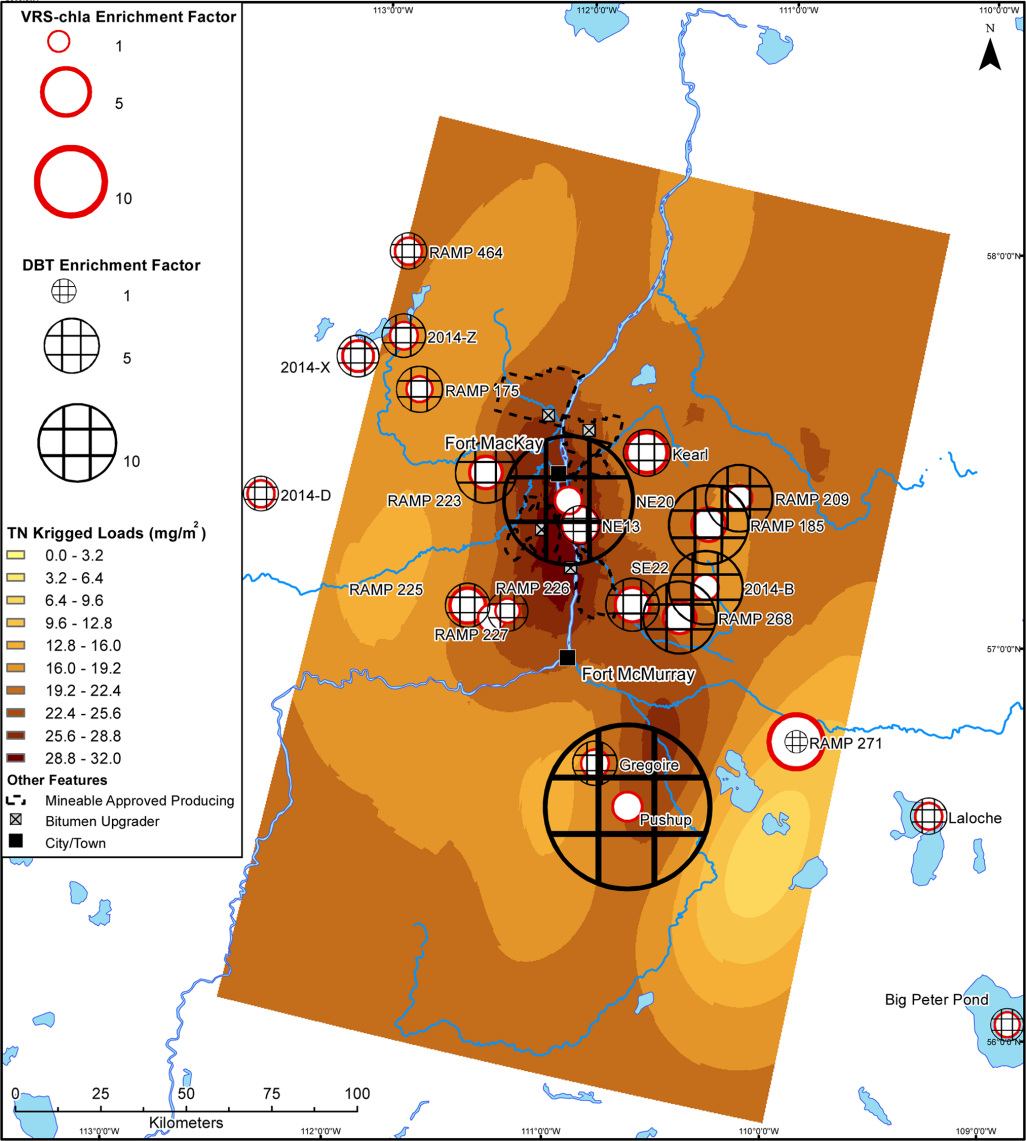
Deposition map of total nitrogen in 2014 snowpack. Interpolated loads of total nitrogen (TN) (mg/m^2^) to the Athabasca Oil Sands Region in March 2014. Sedimentary VRS-chl*a* enrichment factors and DBT enrichment factors from each study lake are overlain.

**Fig 4:**
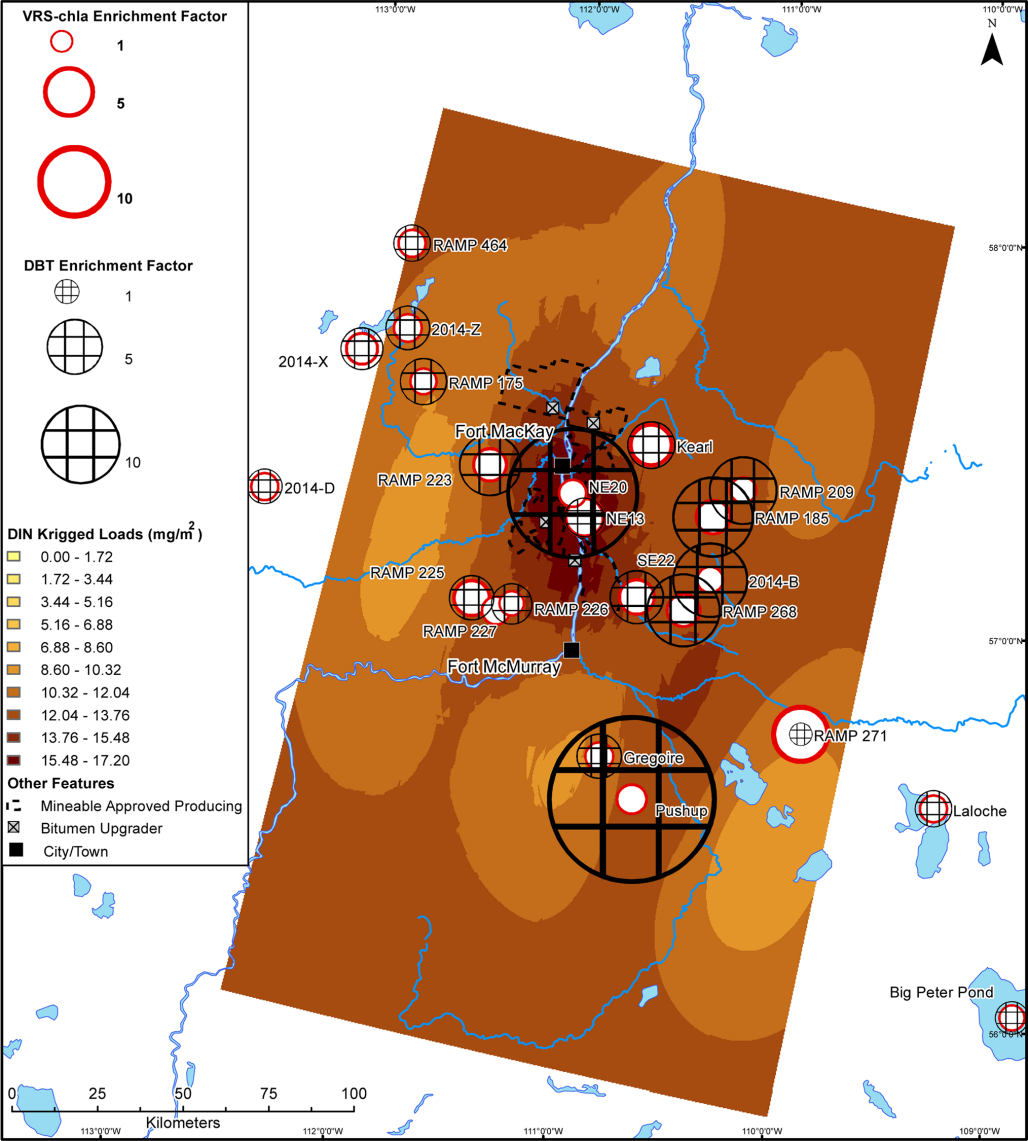
Deposition map of dissolved inorganic nitrogen in 2014 snowpack. Interpolated loads of dissolved inorganic nitrogen (DIN) (mg/m^2^) to the Athabasca Oil Sands Region in March 2014. Sedimentary VRS-chl*a* enrichment factors and DBT enrichment factors from each study lake are overlain.

Maps of interpolated 2014 TN and DIN snowpack loadings show deposition of both total (TN) and biologically-available (DIN) nitrogen, with elevated deposition around the main area of local industrial activity (Figs 3 and 4). This deposition pattern is consistent with that observed for total PACs, unsubstituted PAHs, alkylated PAHs, DBTs, mercury and methyl mercury, several metals known to be emitted in large quantities from the oil sands upgrading facilities (e.g., Ni, V, and Zn), crustal elements (Al and La), and total suspended solids [22]. Using interpolated loadings, we calculated the quantity of nutrients deposited to the area within a 50-km radius of the major oil sands developments. In 2014, deposition of TN and DIN within 50 km of the main area of industrial oil sands development was 172 and 102 tonnes, respectively. In 1978, interpolated loadings of DIN (measures from [46]) also showed elevated deposition around the main industrial area (S6 Fig), only ~15% lower than the 2014 value at 88 tonnes (corrected to account for differences in days of snow accumulation between winter 1977–1978 and winter 2013–2014, 85.5 and 117.5 days respectively). Using these interpolated loadings, we can estimate that at least 3,265 tonnes of DIN has been deposited on the landscape within 50 km of the main industrial oil sands developments.

**Fig 5:**
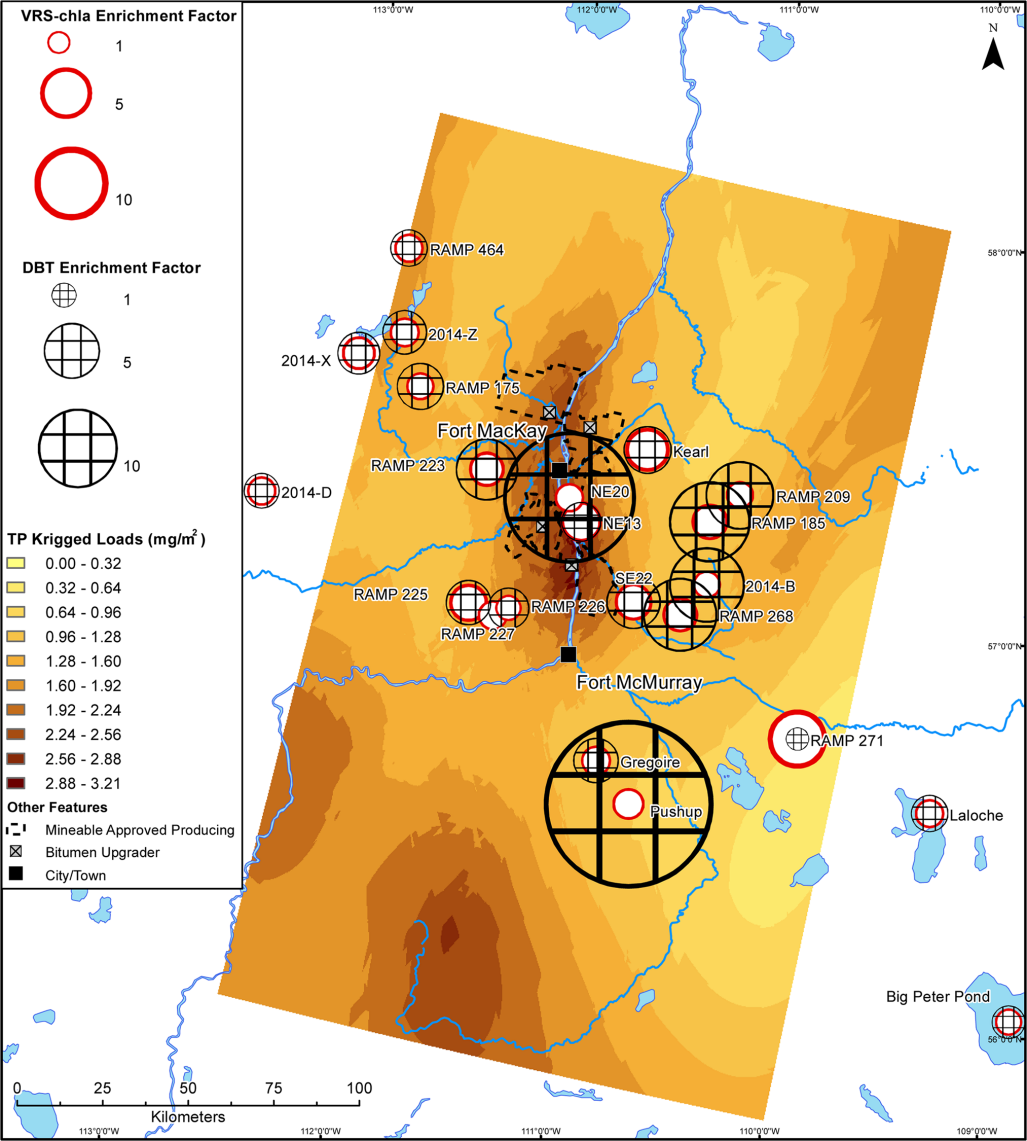
Deposition map of total phosphorus in 2014 snowpack. Interpolated loads of total phosphorus (TP) (mg/m^2^) to the Athabasca Oil Sands Region in March 2014. Sedimentary VRS-chl*a* enrichment factors and DBT enrichment factors from each study lake are overlain.

**Fig 6:**
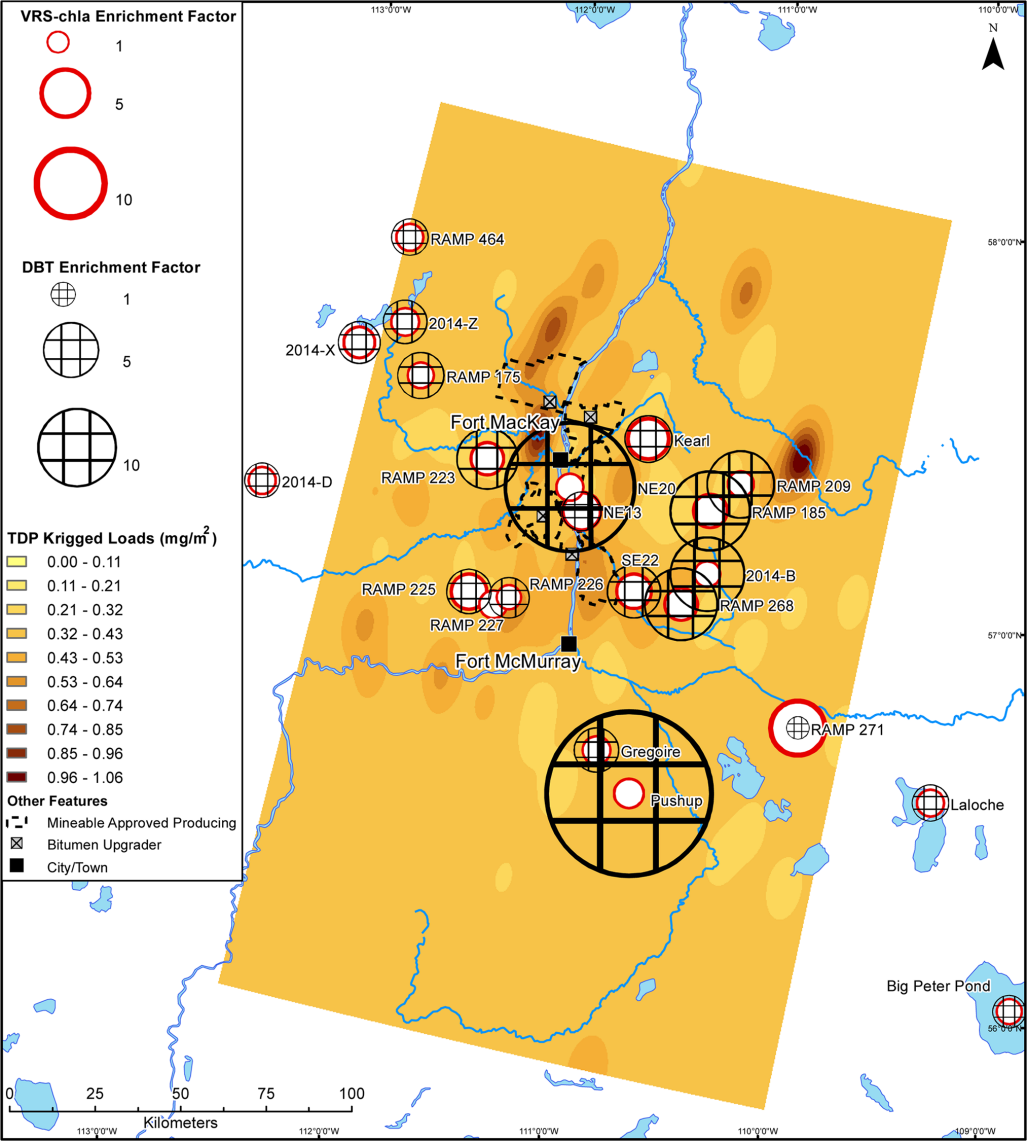
Deposition map of total dissolved phosphorus in 2014 snowpack. Interpolated loads of total dissolved phosphorus (TDP) (mg/m^2^) to the Athabasca Oil Sands Region in March 2014. Sedimentary VRS-chl*a* enrichment factors and DBT enrichment factors from each study lake are overlain.

**Fig 7:**
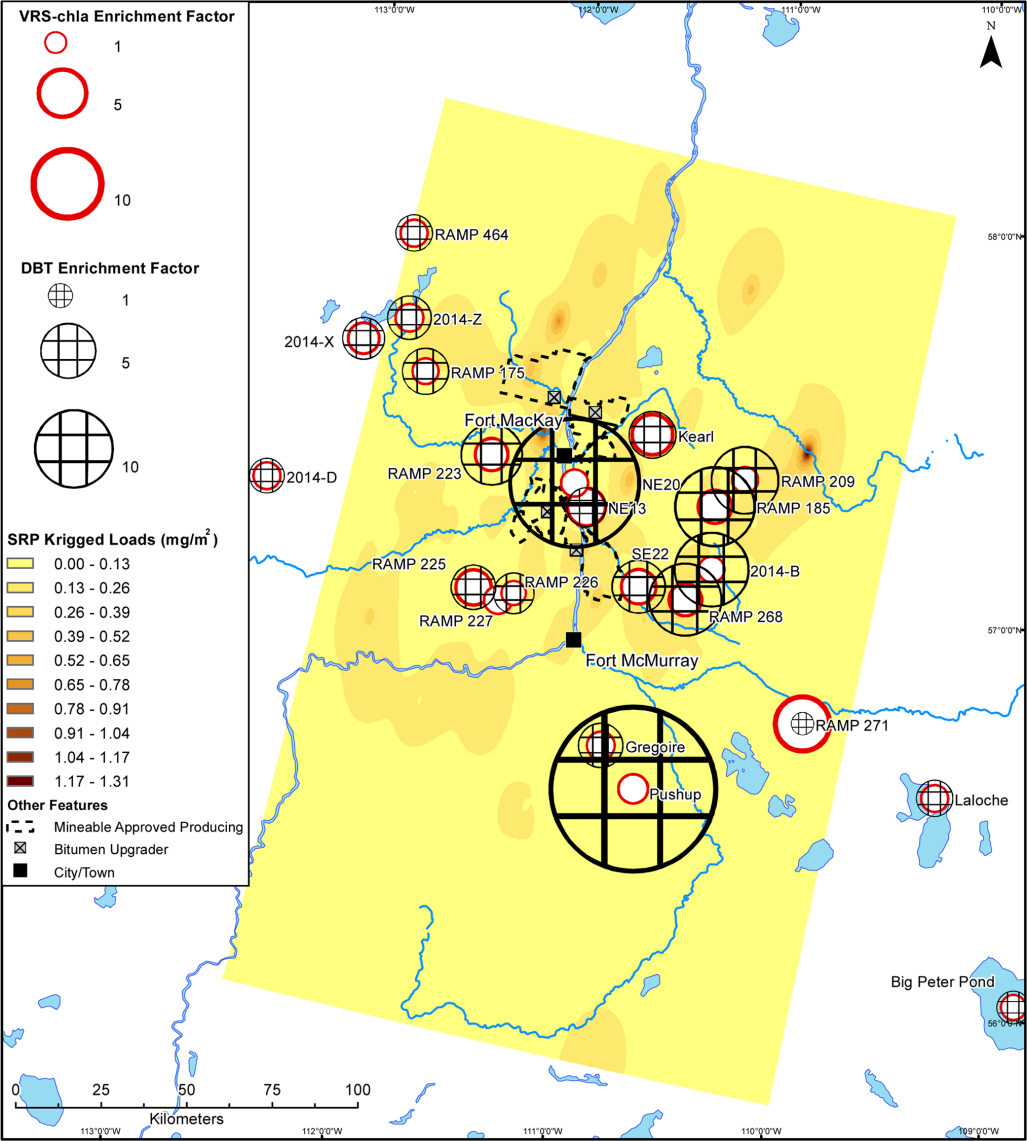
Deposition map of soluble reactive phosphorus in 2014 snowpack. Interpolated loads of soluble reactive phosphorus (SRP) (mg/m^2^) to the Athabasca Oil Sands Region in March 2014. Sedimentary VRS-chl*a* enrichment factors and DBT enrichment factors from each study lake are overlain.

Like TN and DIN, maps of interpolated 2014 spring-time TP across the same area show an industry-centred area of elevated deposition (Fig 5). Conversely, the bioavailable forms of deposited phosphorus (TDP and SRP) are not elevated around the main area of industrial activity (Figs 6 and 7). Total deposition concentrations of TP, TDP, and SRP within 50 km of the main area of oil sands industry in 2014 are 12.0, 2.9, and 1.0 tonnes, respectively.

## Discussion

### Is there a regional increase of primary production in the AOSR?

We show modern primary production greater than background levels at all sites, including those located ~200 km away from the AOSR’s main oil sands developments. Further, there is no relationship between VRS-chl*a* increases and proximity to the main area of development or DBT-inferred industrial oil sands impact. There is also no consistent pattern of VRS-chl*a* increases dependent on landscape position or bioavailable nutrient loading (Figs 4, 6 and 7). Finally, there is no significant difference between average VRS-chl*a* enrichments from minimally and highly DBT-enriched groups. The pervasive, similar increases in VRS-chl*a* concentrations across the AOSR, independent of proximity to industrial development or industrial emissions, suggests a region-wide driver of the enhanced primary production.

### Is there a fertilization effect from upgrading and mining activities?

Our study finds no consistent evidence of nutrient deposition from oil sands industry stimulating increased primary production in industry-impacted lakes. We reason that industry-driven aerial fertilization and associated enhanced primary production would mimic the spatial patterns of bioavailable nutrient deposition (Figs 4, 6 and 7), and/or be most evident within the 50-km zone of high contaminant deposition surrounding the main area of industry [20–22] and diminish with increasing distance [18,19]. Enrichment factors summarize a change over time and enable us to identify an industrial fertilization effect as higher VRS-chla enrichment factors at sites with higher DBT enrichment factors. Our findings suggest no relationship with distance or direction from the centre of industry or DBT enrichment factor, which is independent of distance from the major developments. Nutrient deposition maps based on late winter snow loadings indicate industrial oil sands activities are currently sources of biologically-available nitrogen, but not phosphorus. Although a majority of the study sites are phosphorus-limited lakes, there is no pattern of increased VRS-chl*a* in nitrogen- or phosphorus-limited lakes to match deposition patterns of bioavailable nutrients (Figs 4, 6 and 7). As such, we provide no spatial evidence of industrially-mediated production enrichments in AOSR lakes.

Further, the substantial range in VRS-chl*a* breakpoints from lakes across a DBT-inferred industrial impact gradient (Fig 2, S5 Fig) suggests that primary production is changing on a timeline specific to each lake. Despite this, the majority of lakes (83%) on which breakpoint analyses were applied change after ~1970, suggesting most sites are responding to driver(s) in recent decades. However, the individualistic timing of breakpoints persists in recent decades and the contributions of co-occurring stressors cannot be disentangled using timing of change alone.

Although our findings do not provide consistent spatial or temporal evidence of an industrial fertilizing effect on primary production in lakes across the AOSR, they cannot rule out the threat of continued industrial activities as a potential driver of shifts in lake structure and function.

### What is driving enhanced primary production across the AOSR?

Our findings suggest climate warming as a likely driver of increased aquatic primary production in the AOSR. Significant, positive correlations between average annual and seasonal air temperatures (Table 2), which are increasing in the AOSR (S3 Fig), and standardized VRS-chl*a* concentrations (Z scores) from both groups of DBT-enriched sites (S4 Fig) and all sites combined support the reasoning that a warming climate may be facilitating favourable conditions for primary producers. Specifically, the correlations between standardized VRS-chl*a* concentrations and increased temperatures in the winter, spring, and fall suggest a longer and/or warmer growing season for primary producers as one likely mechanism. Lake ice phenology and stability of the water column are some of the most important controls on primary production and aquatic biological communities in ice-covered lakes [25,28,52], and favourable shifts in light conditions and nutrient availability that accompany an earlier ice-off period are known to stimulate primary production [27]. An increase in the ice-free season of these lakes may be stimulating primary production. Further, warming temperatures, in scenarios where there are no other limiting resources, typically yield increased primary production [53].

Our findings indicate that the variable, decadal-scale precipitation patterns in the AOSR are not playing a significant role in driving increased aquatic primary production. Although increases in primary production from our 23 lakes are not significantly correlated to precipitation (S6 Table), reduced moisture due to periods of low precipitation, reduced snowpack and ice-cover, and increased evaporation caused by warmer temperatures [9] can concentrate nutrients and cations in waterbodies [28,29,32] and could exacerbate production trends, especially in shallow lakes. The larger, though still not significant, correlation coefficients (r) between VRS-chl*a* and winter precipitation and VRS-chl*a* and fall precipitation suggest that snow may be an important component of the relationship between total precipitation and primary production in the AOSR. Further, shallow lakes with small catchments, which form the majority of our study sites, may not be reflecting changes in precipitation inflow to the same degree as would larger, deeper lakes with substantial catchments and inflows.

Our findings of significant correlations to warming temperatures are congruous with findings from biological species assemblages demonstrating changes consistent with recent regional climate warming in lakes near (within 50 km) [15] AOSR industry and further downwind (up to 300 km NE) [17]. The consistent findings of recent AOSR studies, including our study presented here, provide strong evidence of complex multiple-stressor systems in which climate warming plays an important role.

### Summary and future directions

Our study’s inclusion of many diverse lakes located up to ~200 km away from the centre of industrial oil sands activities facilitates assessment of broad-scale patterns and development in our understanding of the AOSR in a dual scenario of climate change and intense industrial activity. Our findings confirm a regional increase in primary production and indicate that warming temperatures are an important driver in AOSR aquatic primary production. Improved understanding of the roles and relative importance of industrial development and climatic change on foundational ecosystem processes in the AOSR likely lies in the assessment of downcore biotic and abiotic (e.g., stable nitrogen and carbon isotope) trends. These analyses should further elucidate the production histories and nutrient sources of regional lakes, and provide crucial information regarding the vulnerability of these aquatic systems.

## Supporting Information

S1 Table Lake Names. Names and alternate names for study sites. Asterisks (*) denote names that have been previously used in academic literature.
(TIF)

S2 Table Selected physical and chemical parameters of study lakes. (TIF)

S3 Table Summary of kriging settings. ArcGIS10 Geostatistical Analyst software settings used to generate deposition maps for dissolved inorganic nitrogen (DIN) 1978, DIN 2014, total nitrogen (TN) 2014, soluble reactive phosphorus (SRP) 2014, total dissolved phosphorus (TDP) 2014, and total phosphorus (TP) 2014.
(TIF)

S4 Table Estimated spatial extent of nutrient deposition. Estimated spatial extent (km^2^) of interpolated spring-time snowpack loadings (mg/g^2^) of dissolved inorganic nitrogen (DIN) 1978, DIN 2014, total nitrogen (TN) 2014, soluble reactive phosphorus (SRP) 2014, total dissolved phosphorus (TDP) 2014, and total phosphorus (TP) 2014 obtained by geostatistical interpolation of measured spring-time snowpack loadings using ArcGIS Geostatistical Analyst software.
(TIF)

S5 Table Comparisons of kriging range means and measured means. Kriging means, measured means, and differences between kriged and measured means in each kriging zone for dissolved inorganic nitrogen (DIN) 1978, DIN 2014, total nitrogen (TN) 2014, soluble reactive phosphorus (SRP) 2014, total dissolved phosphorus (TDP) 2014, and total phosphorus (TP) 2014.
(PDF)

S6 Table Correlations between 5-year averaged VRS-chl*a* Z scores and 5-year averaged precipitation data. Results from Pearson correlations between VRS-chl*a* Z scores (averaged over 5-year intervals) from highly and minimally DBT-enriched lakes and annual and seasonal AOSR precipitation (averaged over the same 5-year intervals).
(TIF)

S1 Fig Sediment core dating. Downcore profiles of **s**upported (^226^Ra activity) (blue dashed line) and total ^210^Pb activity (red circles). Downcore profiles of ^137^Cs activity (yellow triangles) (± 1 SD), and age-depth models (black and grey circles) for 23 sediment cores (A-W). Black circles represent constant rate of supply (CRS)-inferred dates. Grey circles represent extrapolated dates. Age-depth models were developed using the depth midpoint of sediment intervals, the CRS-inferred age, and polynomial regression (second, third, or fourth-order) with intercept set to the time of coring. The yellow star overlain on the CRS dates denotes the depth of the 1963 ^137^Cs peak. Profiles are ordered by year of core collection.
(PDF)

S2 Fig Snowpack sample sites. Maps of sites in the Athabasca Oil Sands Region where snowpack samples were collected in January 1978 and March 2014.
(TIF)

S3 Fig 5-year averages of mean annual and seasonal temperature and precipitation data. Historical temperature (station no. 3062696) and precipitation (station no. 3062693) data for Fort McMurray obtained from Environment Canada’s Adjusted and Homogenized Canadian Climate Data website (www.ec.gc.ca/dccha-ahccd) dating back to 1916 and 1920, respectively.
(TIF)

S4 Fig 5-year averages of VRS-chl*a* Z scores. 5-year average VRS-chla Z scores from (A) minimally and (B) highly DBT-enriched sites.
(TIF)

S5 Fig VRS-chl*a* profiles. Downcore VRS-chl*a* concentrations for each site calibrated to include diagenetic processes arranged by DBT enrichment factor. (A) Profiles and breakpoints for highly DBT-enriched lakes suitable for breakpoint analysis (n = 13), (B) profiles and breakpoints for minimally DBT-enriched lakes suitable for breakpoint analysis (n = 5), and (C) profiles for minimally (Big Peter Pond), highly (RAMP 175, RAMP 226, and 2014-B), and undetermined (RAMP 227) DBT-enriched lakes where breakpoint analysis is not applicable. RAMP 175, RAMP 226, and 2014-B do not demonstrate stable baselines, Big Peter Pond has one extreme point in recent sediments that erroneously impacts timing of a breakpoint, and RAMP 227 does not have a long enough DBT dataset to calculate a DBT-enrichment factor.
(PDF)

S6 Fig Deposition map of dissolved inorganic nitrogen in 1978 snowpack. Interpolated loads of dissolved inorganic nitrogen (DIN) (mg/m^2^) to the Athabasca Oil Sands Region in January 1978.
(TIF)
