# Supplementary figures and images for: Recent Warming, Rather than Industrial Emissions of Bioavailable Nutrients, Is the Dominant Driver of Lake Primary Production Shifts across the Athabasca Oil Sands Region
Source: PLoS One. 2016 May 2;11(5):e0153987. doi: 10.1371/journal.pone.0153987 (PMC4852901; doi:10.1371/journal.pone.0153987)

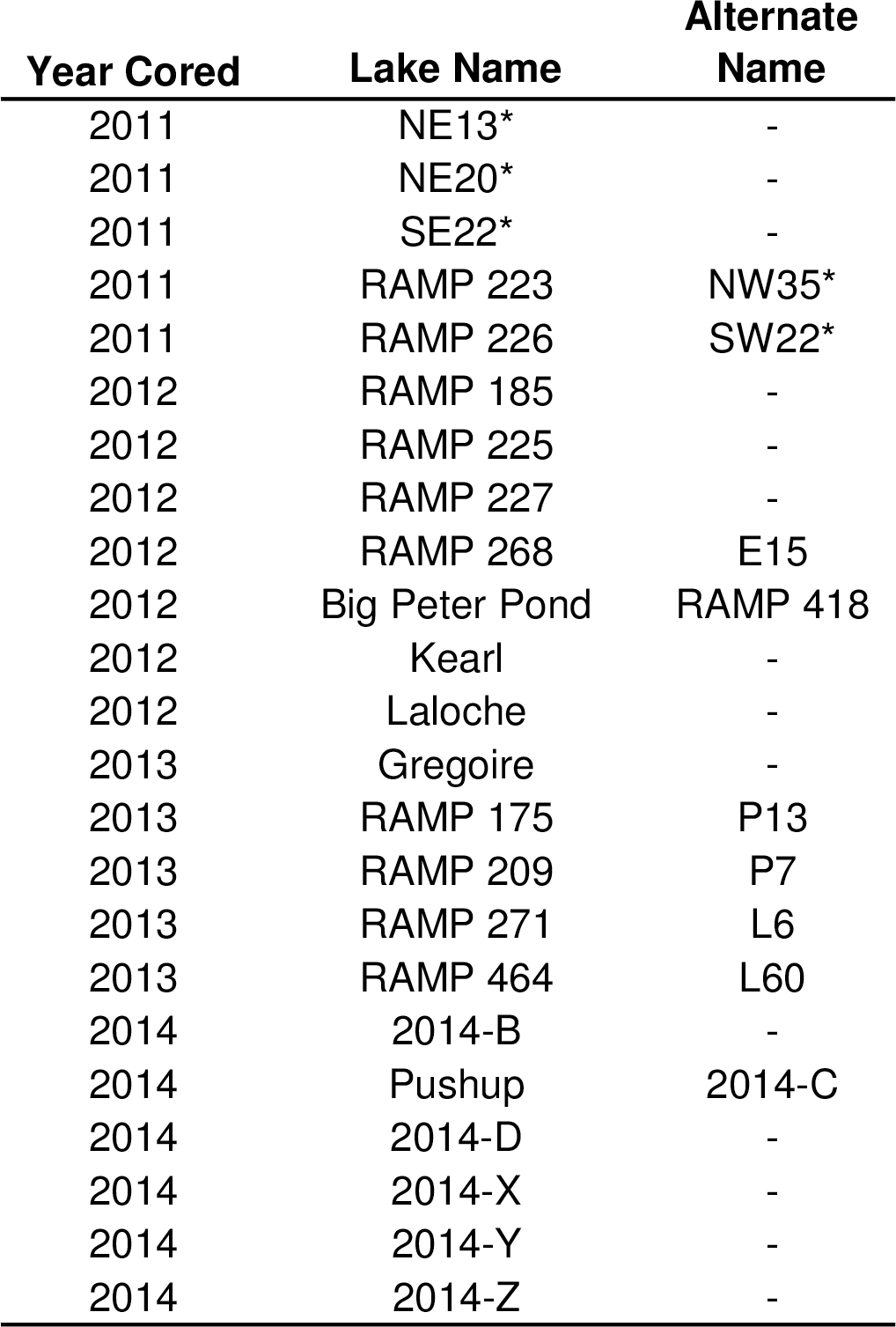

Supplement: S1 Table — Names and alternate names for study sites. Asterisks (*) denote names that have been previously used in academic literature. (TIF) [file pone.0153987.s001.tif]

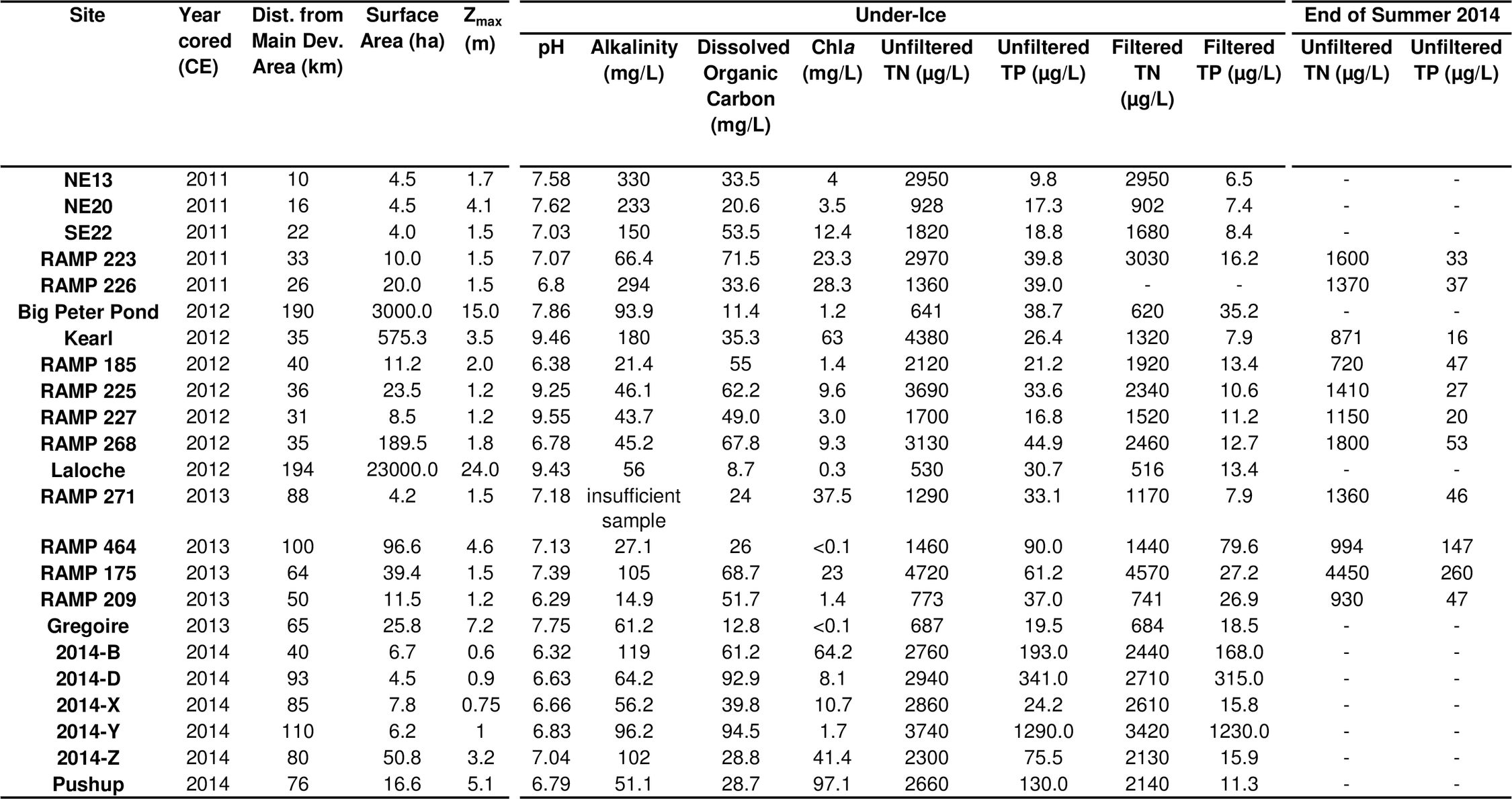

Supplement: S2 Table — (TIF) [file pone.0153987.s002.tif]

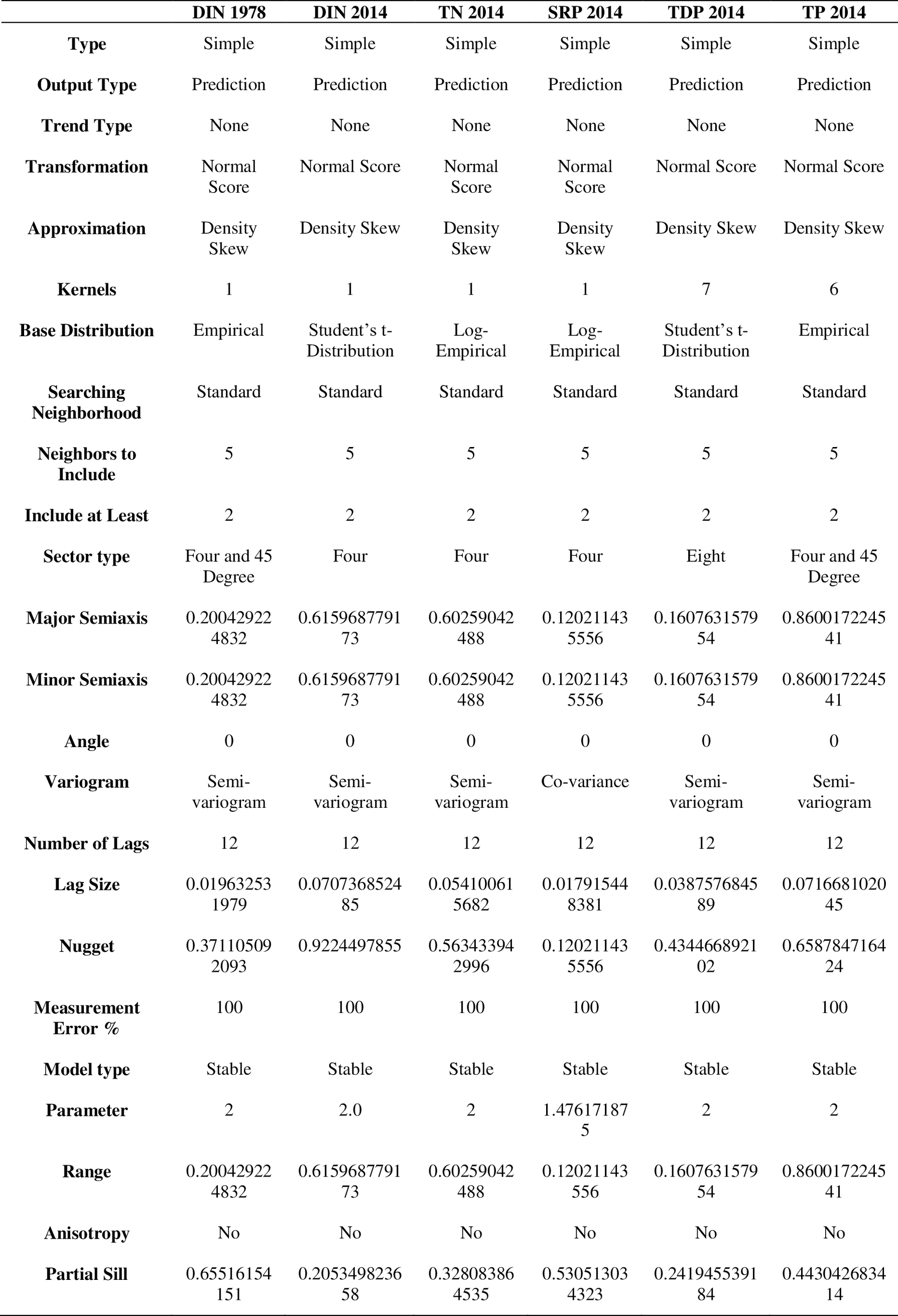

Supplement: S3 Table — ArcGIS10 Geostatistical Analyst software settings used to generate deposition maps for dissolved inorganic nitrogen (DIN) 1978, DIN 2014, total nitrogen (TN) 2014, soluble reactive phosphorus (SRP) 2014, total dissolved phosphorus (TDP) 2014, and total phosphorus (TP) 2014. (TIF) [file pone.0153987.s003.tif]

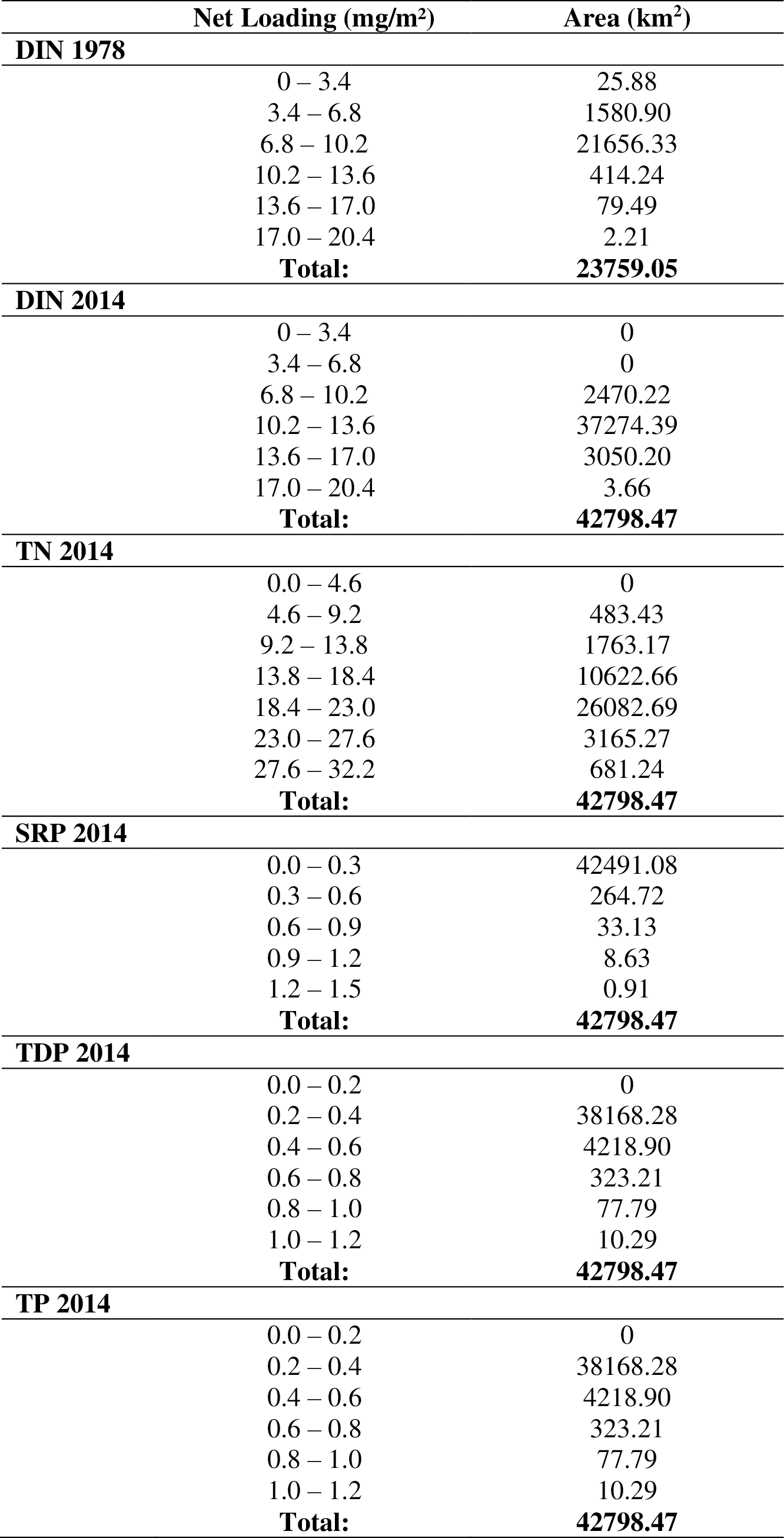

Supplement: S4 Table — Estimated spatial extent (km2) of interpolated spring-time snowpack loadings (mg/g2) of dissolved inorganic nitrogen (DIN) 1978, DIN 2014, total nitrogen (TN) 2014, soluble reactive phosphorus (SRP) 2014, total dissolved phosphorus (TDP) 2014, and total phosphorus (TP) 2014 obtained by geostatistical interpolation of measured spring-time snowpack loadings using ArcGIS Geostatistical Analyst software. (TIF) [file pone.0153987.s004.tif]

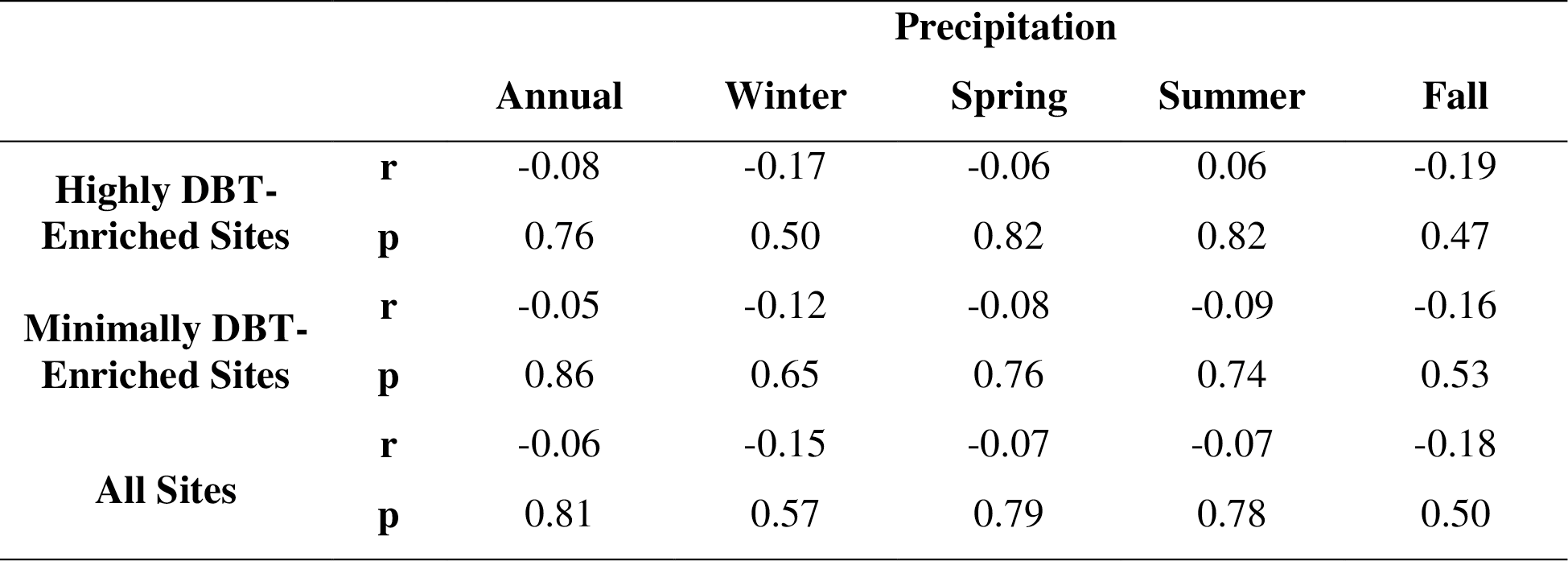

Supplement: S6 Table — Results from Pearson correlations between VRS-chla Z scores (averaged over 5-year intervals) from highly and minimally DBT-enriched lakes and annual and seasonal AOSR precipitation (averaged over the same 5-year intervals). (TIF) [file pone.0153987.s006.tif]

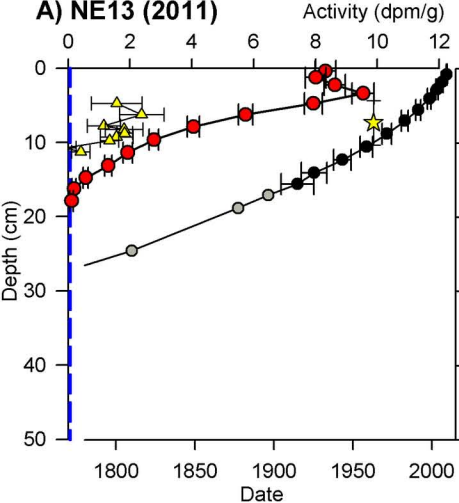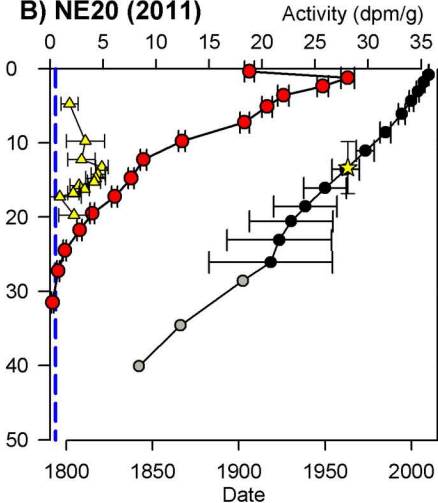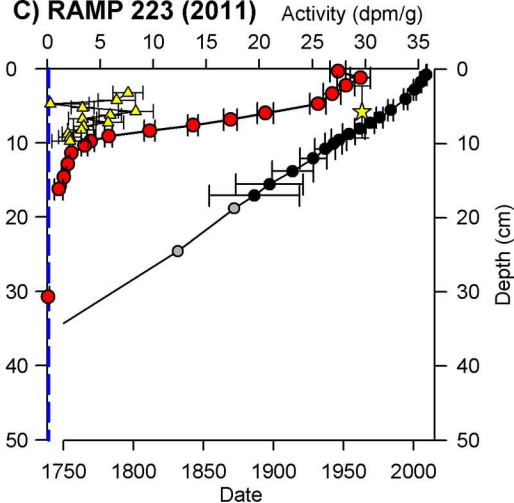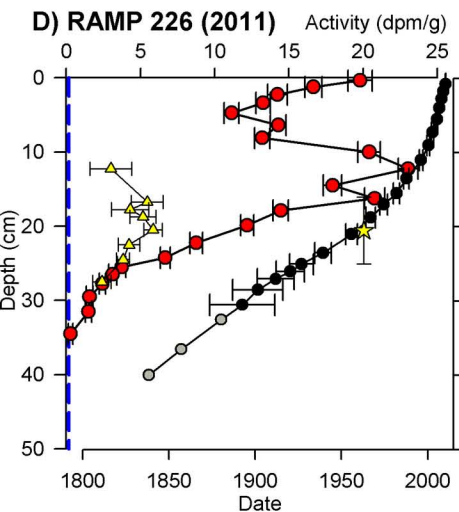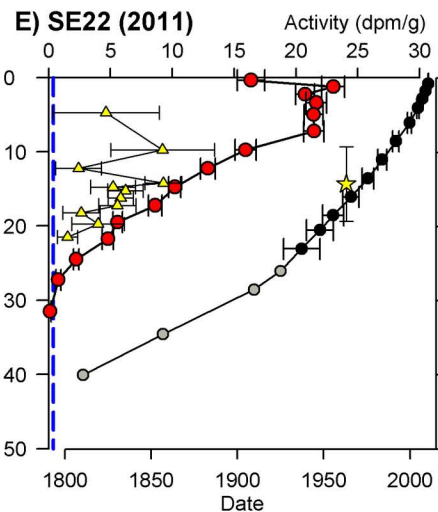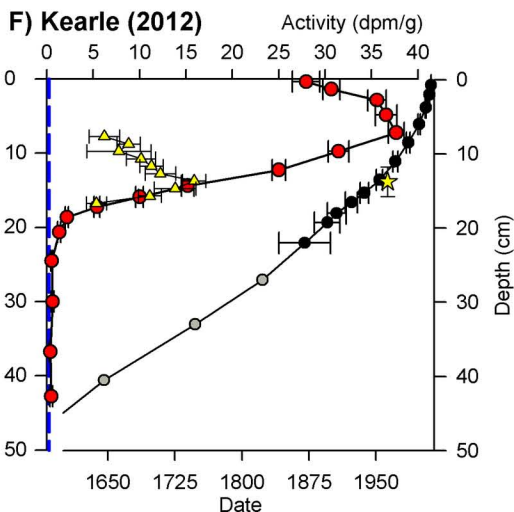

**G) RAMP 185 (2012)**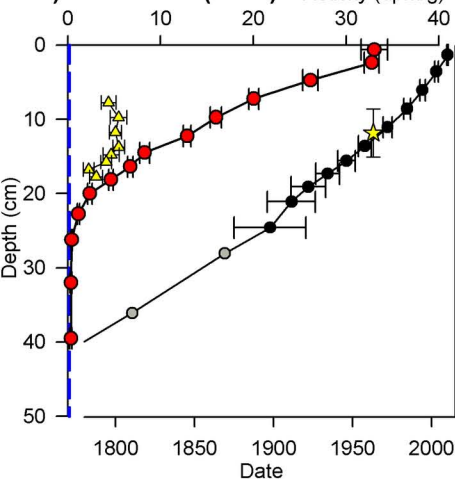**H) RAMP 225 (2012)**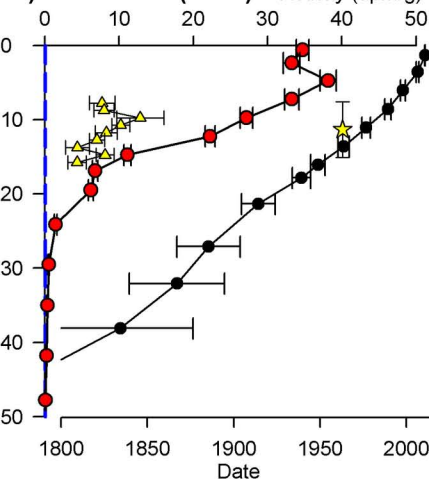**I) RAMP 227 (2012)**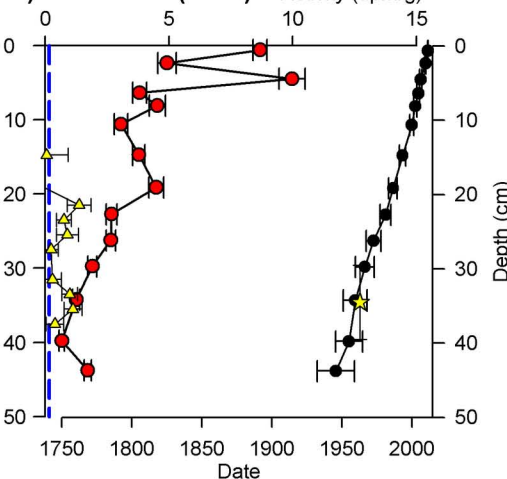**J) RAMP 268 (2012)**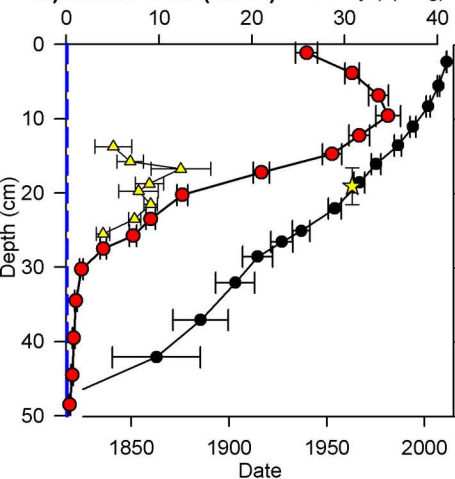**K) Laloche (2012)**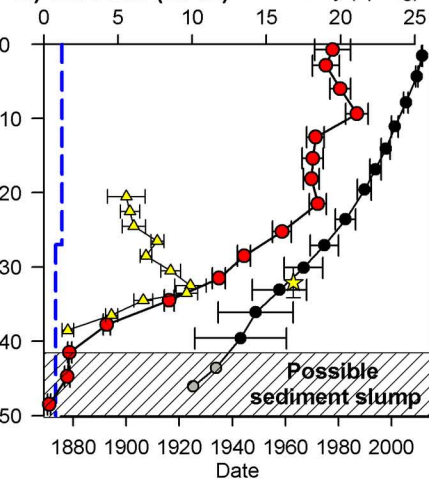**L) Big Peter Pond (2012)**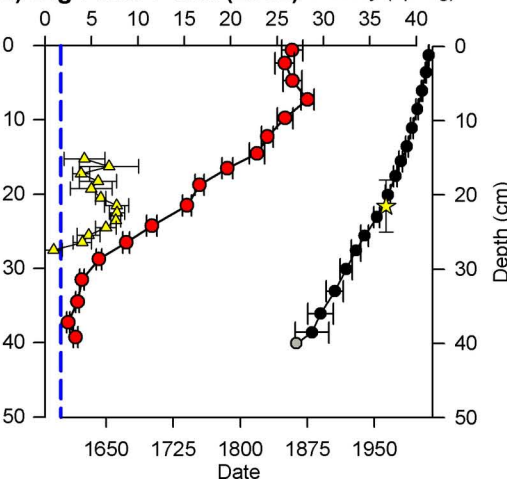

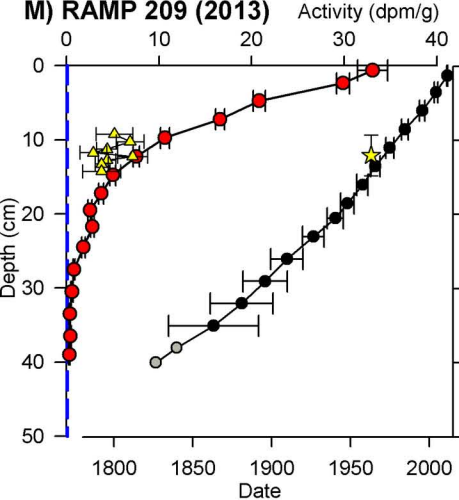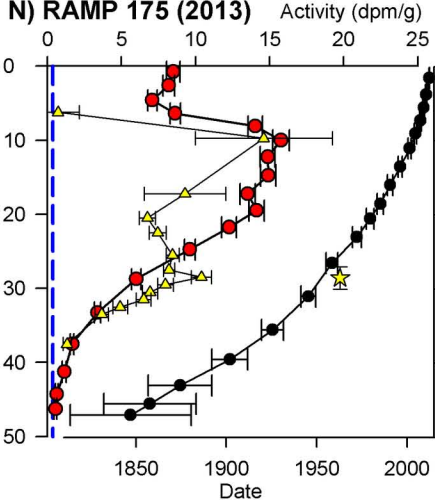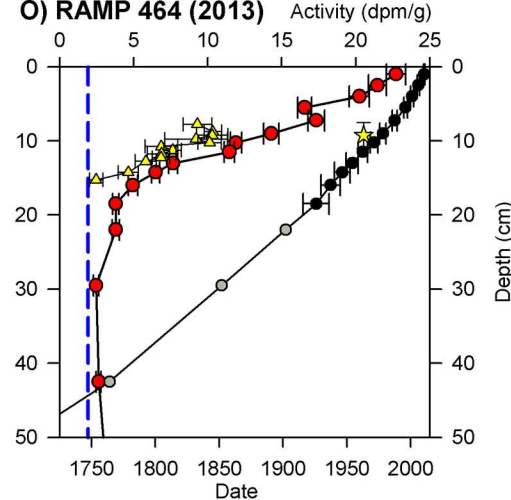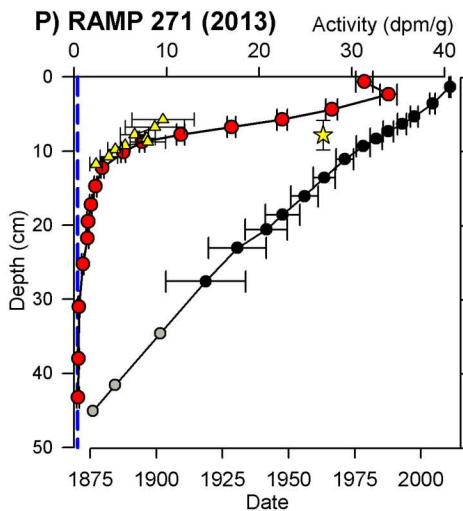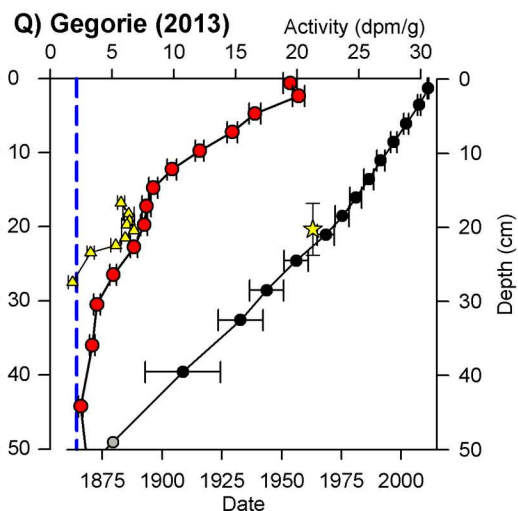

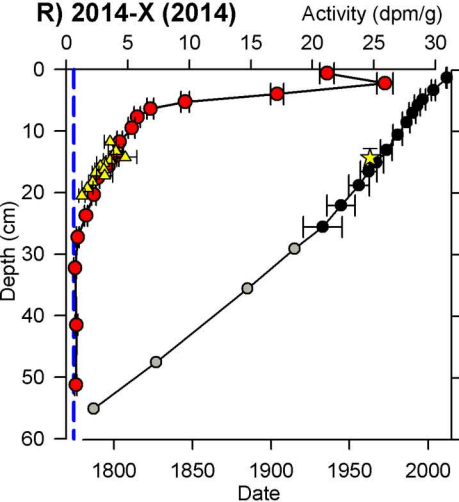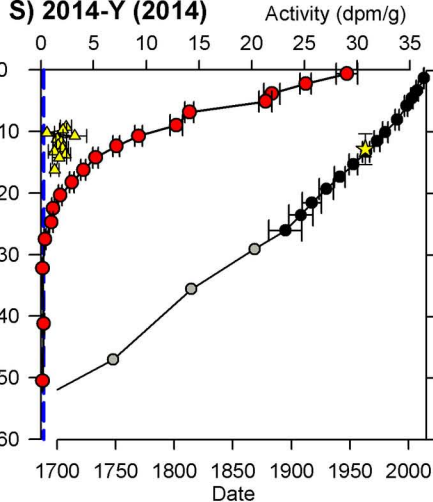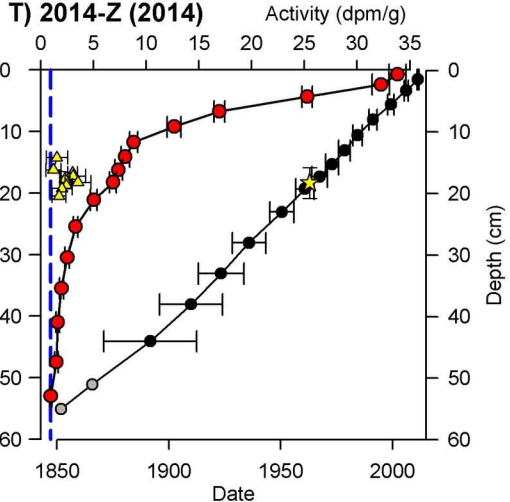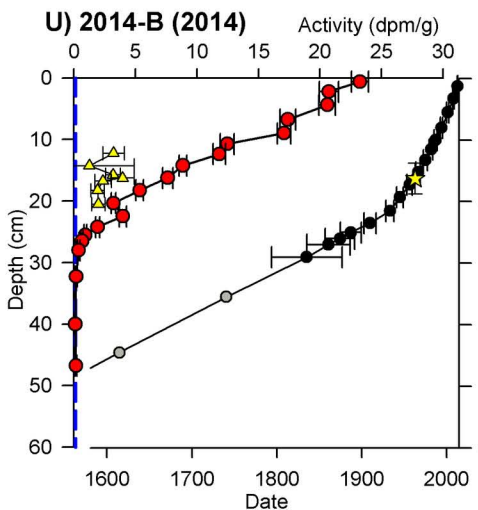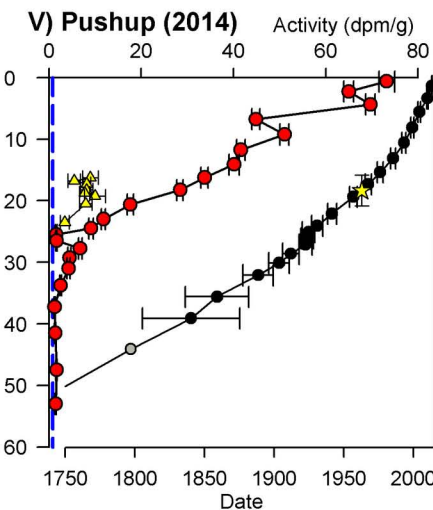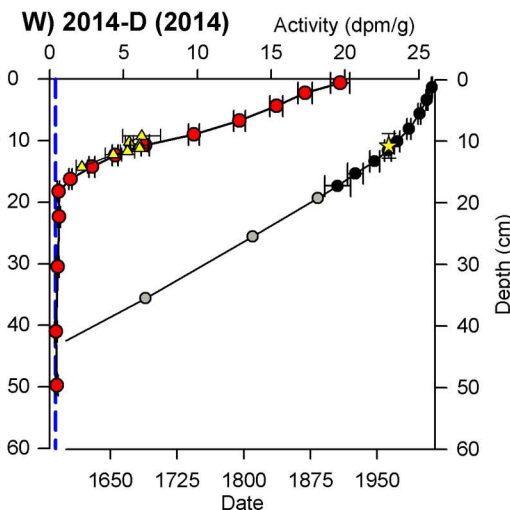

Supplement: S1 Fig — Downcore profiles of supported (226Ra activity) (blue dashed line) and total 210Pb activity (red circles). Downcore profiles of 137Cs activity (yellow triangles) (± 1 SD), and age-depth models (black and grey circles) for 23 sediment cores (A-W). Black circles represent constant rate of supply (CRS)-inferred dates. Grey circles represent extrapolated dates. Age-depth models were developed using the depth midpoint of sediment intervals, the CRS-inferred age, and polynomial regression (second, third, or fourth-order) with intercept set to the time of coring. The yellow star overlain on the CRS dates denotes the depth of the 1963 137Cs peak. Profiles are ordered by year of core collection. (PDF) [file pone.0153987.s007.pdf]

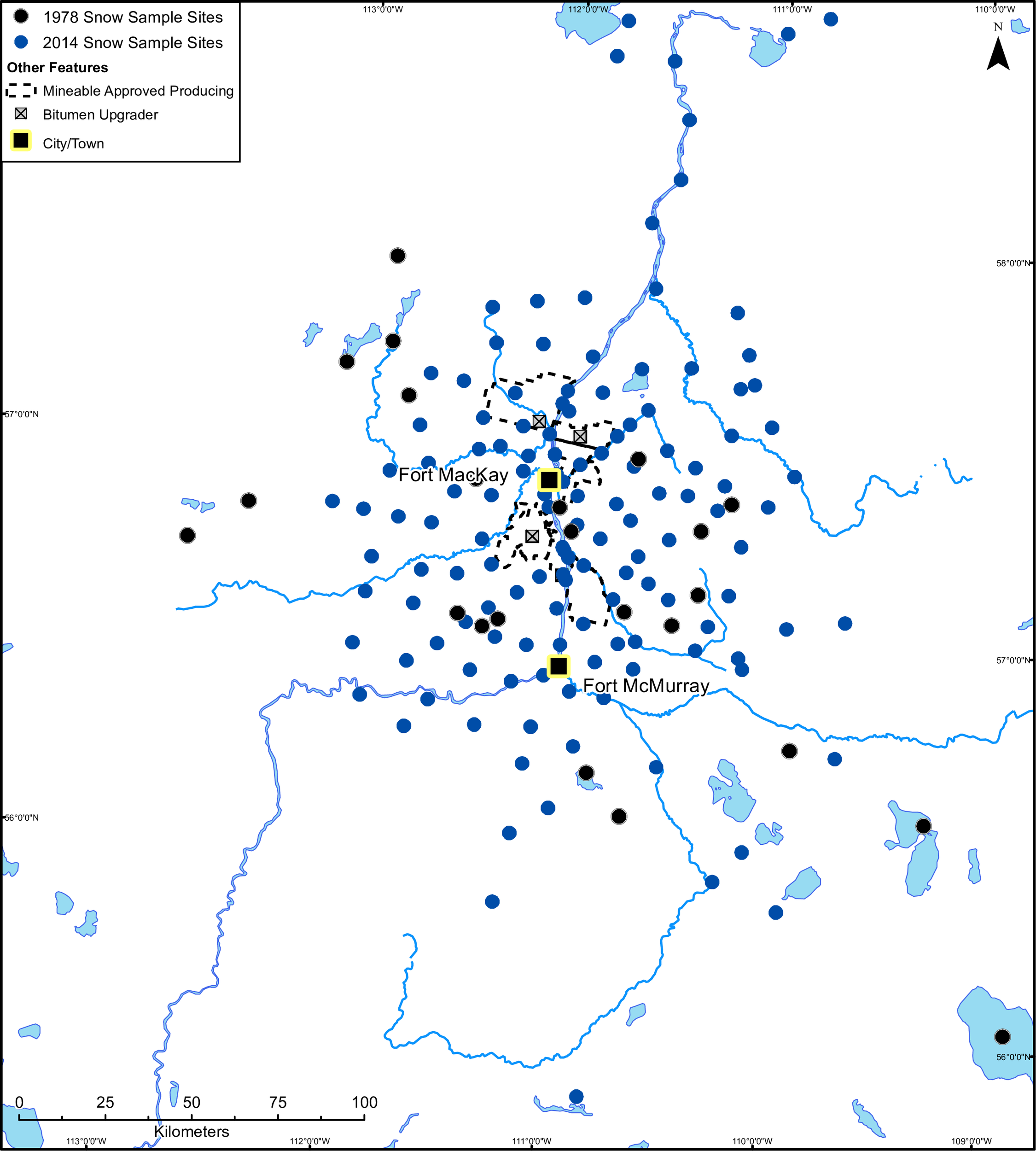

Supplement: S2 Fig — Maps of sites in the Athabasca Oil Sands Region where snowpack samples were collected in January 1978 and March 2014. (TIF) [file pone.0153987.s008.tif]

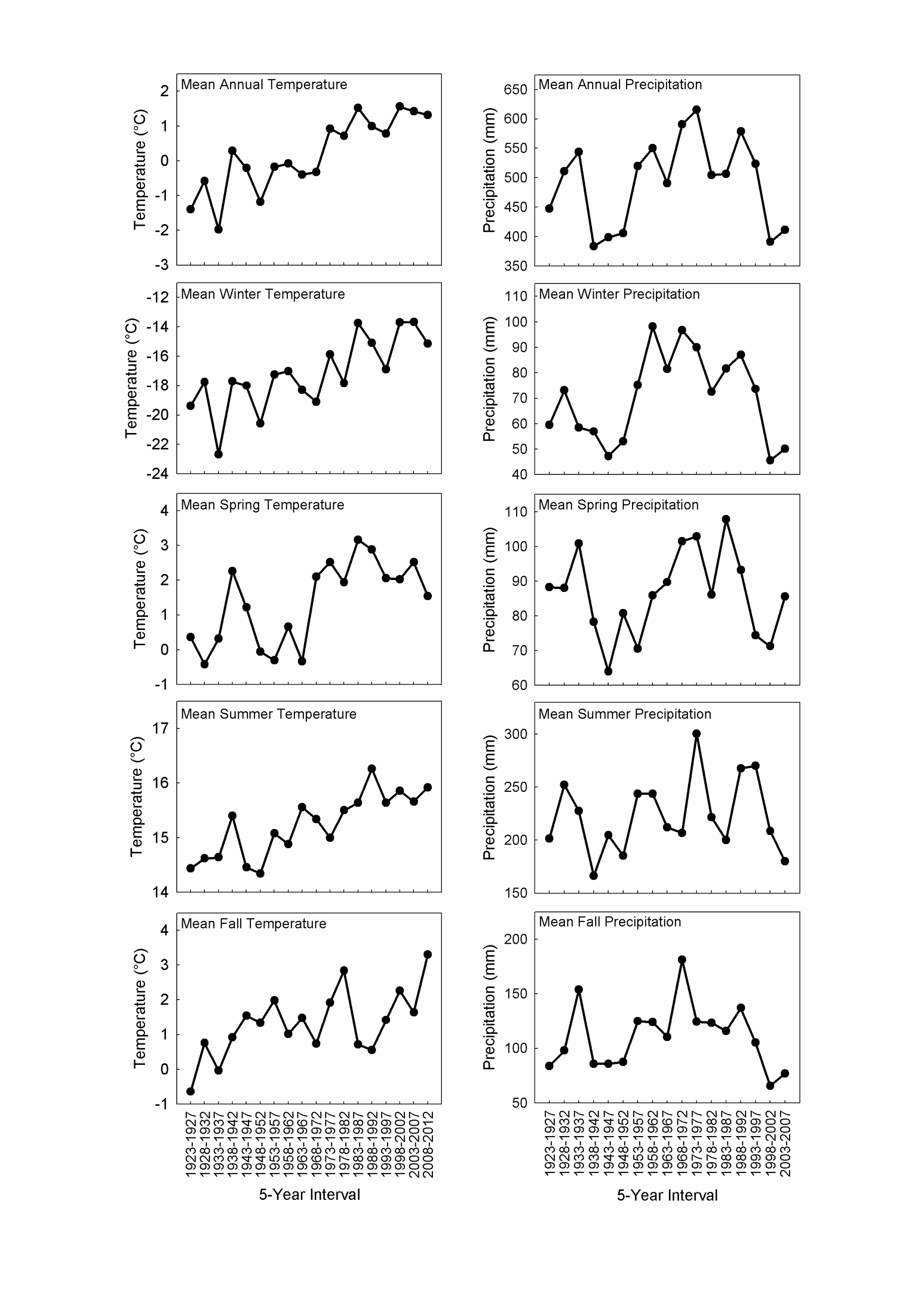

Supplement: S3 Fig — Historical temperature (station no. 3062696) and precipitation (station no. 3062693) data for Fort McMurray obtained from Environment Canada’s Adjusted and Homogenized Canadian Climate Data website (www.ec.gc.ca/dccha-ahccd) dating back to 1916 and 1920, respectively. (TIF) [file pone.0153987.s009.tif]

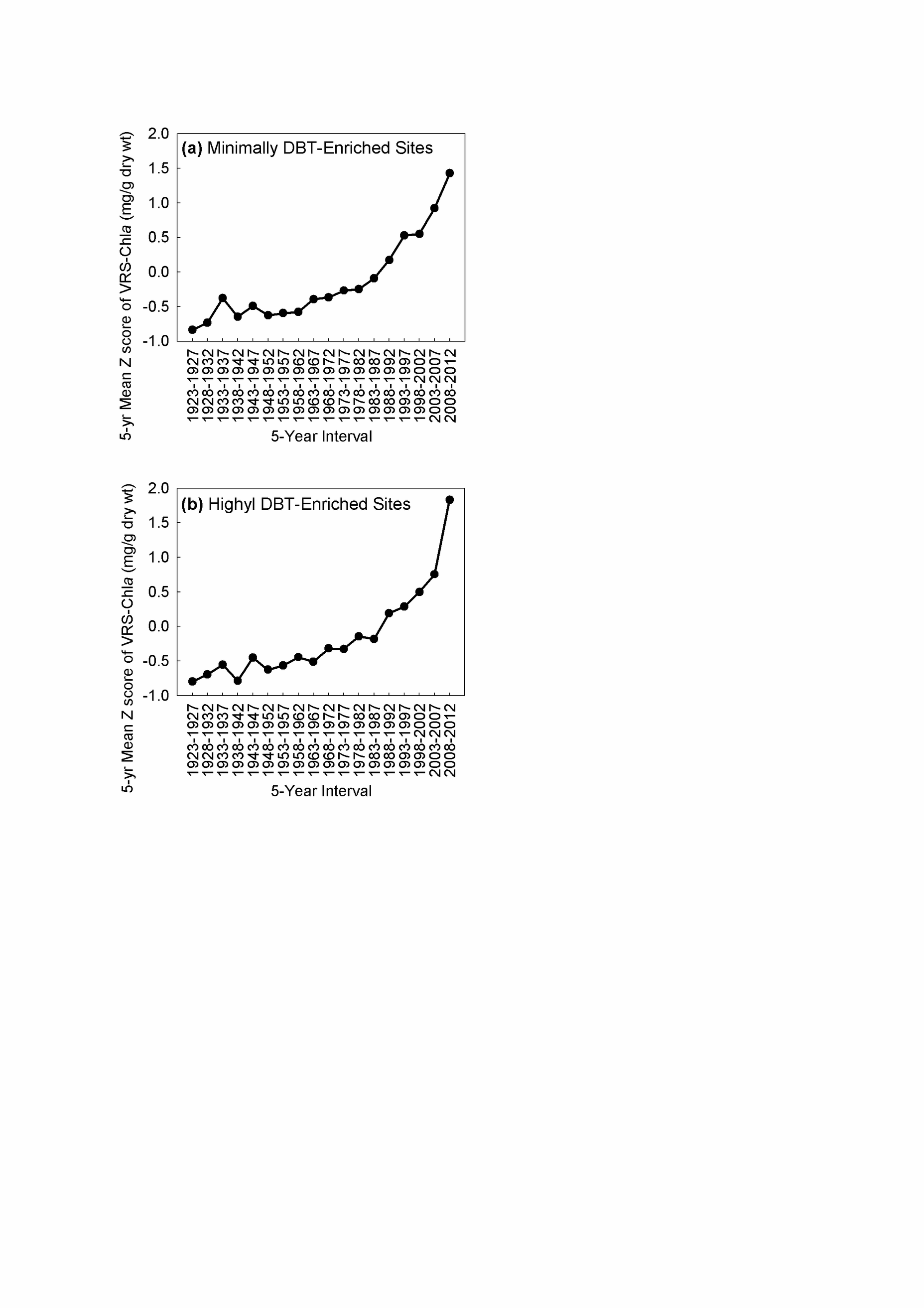

Supplement: S4 Fig — 5-year average VRS-chla Z scores from (A) minimally and (B) highly DBT-enriched sites. (TIF) [file pone.0153987.s010.tif]

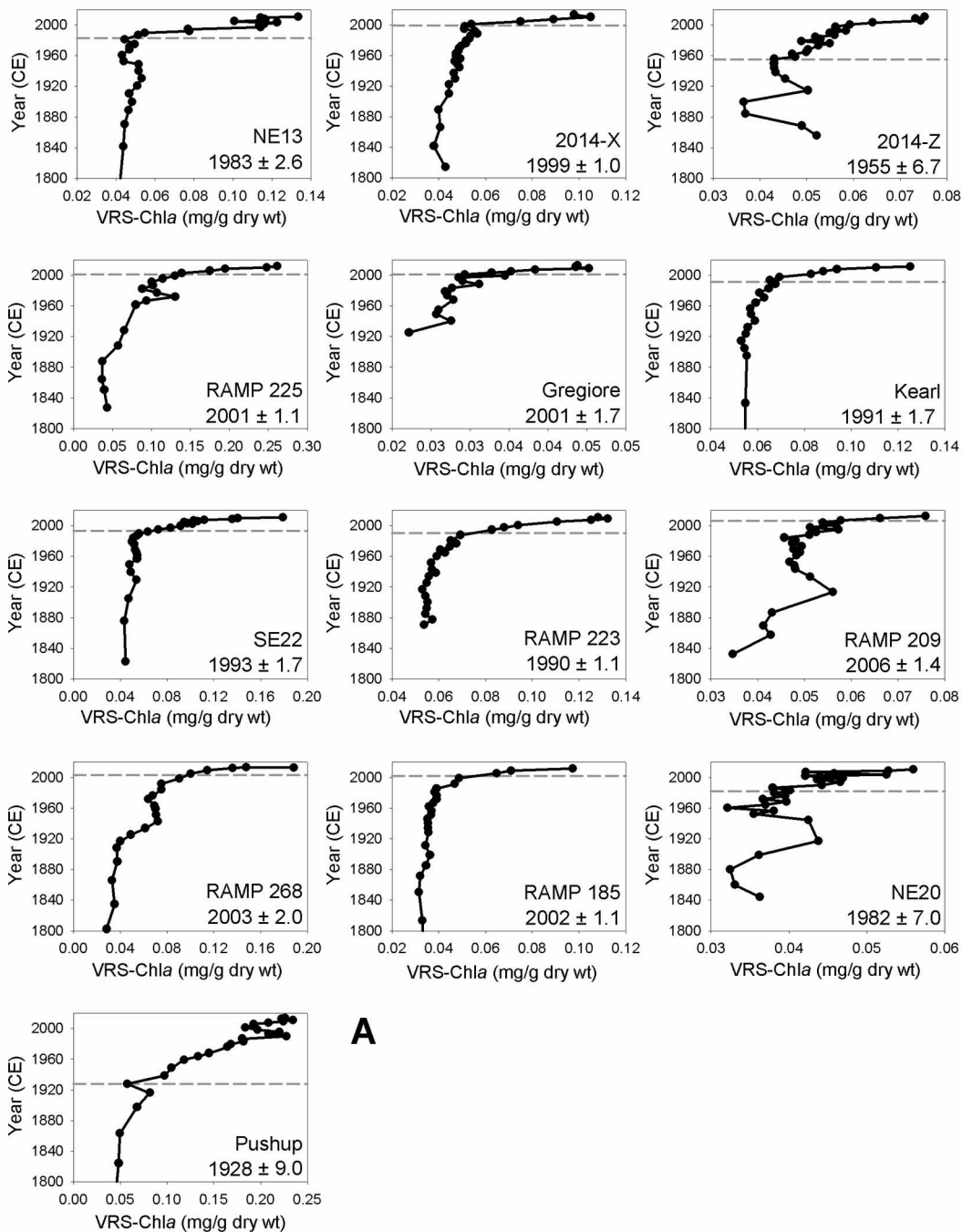

**A**

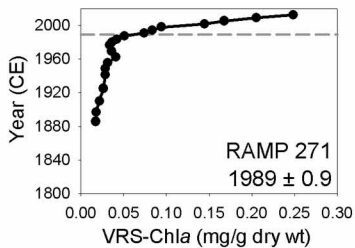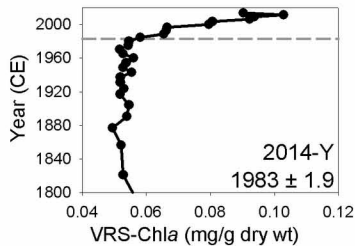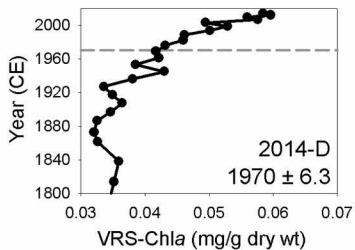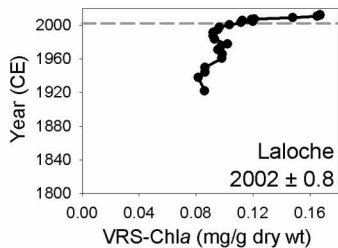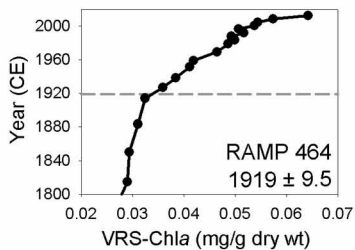

**B**

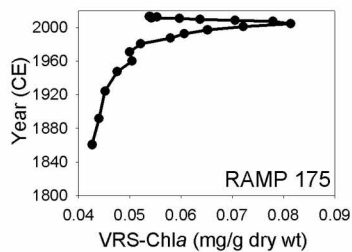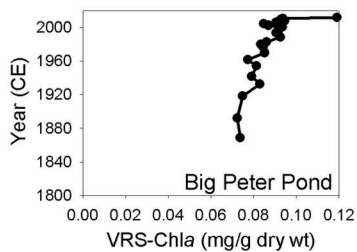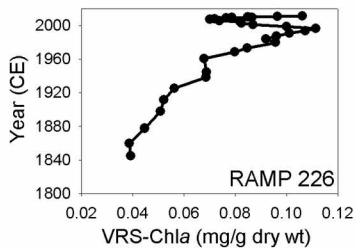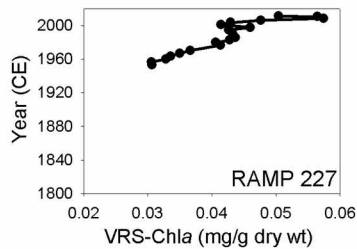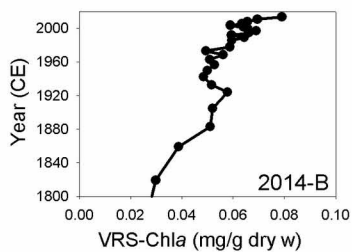

C

Supplement: S5 Fig — Downcore VRS-chla concentrations for each site calibrated to include diagenetic processes arranged by DBT enrichment factor. (A) Profiles and breakpoints for highly DBT-enriched lakes suitable for breakpoint analysis (n = 13), (B) profiles and breakpoints for minimally DBT-enriched lakes suitable for breakpoint analysis (n = 5), and (C) profiles for minimally (Big Peter Pond), highly (RAMP 175, RAMP 226, and 2014-B), and undetermined (RAMP 227) DBT-enriched lakes where breakpoint analysis is not applicable. RAMP 175, RAMP 226, and 2014-B do not demonstrate stable baselines, Big Peter Pond has one extreme point in recent sediments that erroneously impacts timing of a breakpoint, and RAMP 227 does not have a long enough DBT dataset to calculate a DBT-enrichment factor. (PDF) [file pone.0153987.s011.pdf]

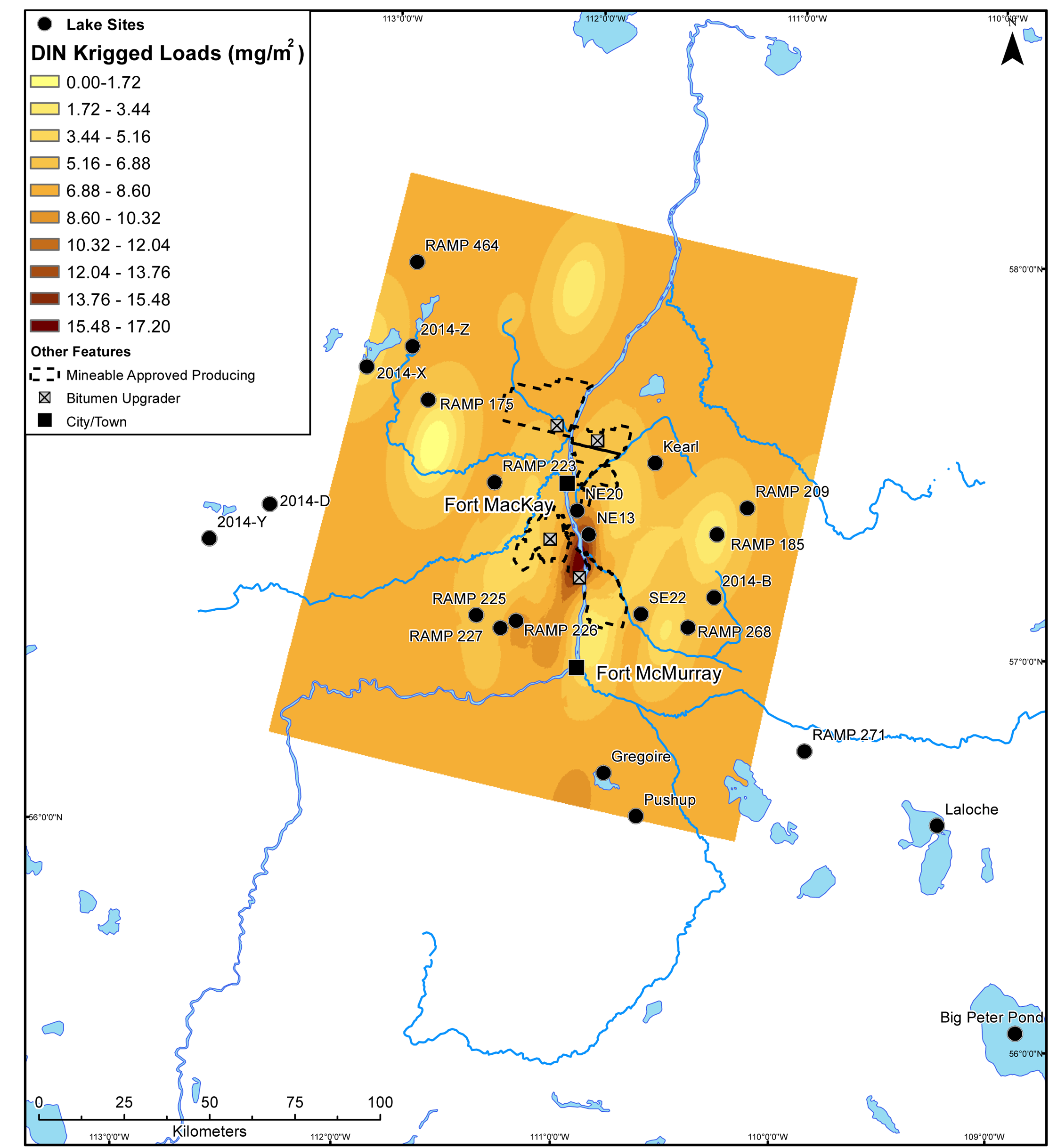

Supplement: S6 Fig — Interpolated loads of dissolved inorganic nitrogen (DIN) (mg/m2) to the Athabasca Oil Sands Region in January 1978. (TIF) [file pone.0153987.s012.tif]
