# Supplementary material for: Recent Warming, Rather than Industrial Emissions of Bioavailable Nutrients, Is the Dominant Driver of Lake Primary Production Shifts across the Athabasca Oil Sands Region
Source: PLoS One. 2016 May 2;11(5):e0153987. doi: 10.1371/journal.pone.0153987 (PMC4852901; doi:10.1371/journal.pone.0153987)
Supplement: S5 Table — Kriging means, measured means, and differences between kriged and measured means in each kriging zone for dissolved inorganic nitrogen (DIN) 1978, DIN 2014, total nitrogen (TN) 2014, soluble reactive phosphorus (SRP) 2014, total dissolved phosphorus (TDP) 2014, and total phosphorus (TP) 2014. (PDF) [file pone.0153987.s005.pdf]

	Kriging Range (mg/m²)	Kriging Range Mean (mg/m²)	Sampled Mean (mg/m²)	Difference from Sampled Mean
**DIN 1978**				
	0.0 – 3.4	1.7	0	1.7
	3.4 – 6.8	5.1	4.4	0.7
	6.8 – 10.2	8.5	8.3	0.2
	10.2 – 13.6	11.9	12.6	-0.7
	13.6 – 17.0	15.3	17.9	-2.6
	17.0 – 20.4	18.7	0	18.7
	20.4 – 23.8	22.1	0	22.1
	23.8 – 27.2	25.5	0	25.5
	27.2 – 30.6	28.9	0	28.9
	30.6 – 34.0	32.3	0	32.3
**DIN 2014**				
	0 – 3.4	1.7	0	1.7
	3.4 – 6.8	5.1	0	5.1
	6.8 – 10.2	8.5	4.5	4.0
	10.2 – 13.6	11.9	11.6	0.3
	13.6 – 17.0	15.3	17.9	-2.6
	17.0 – 20.4	18.7	0	18.7
	20.4 – 23.8	22.1	0	22.1
	23.8 – 27.2	25.5	0	25.5
	27.2 – 30.6	28.9	0	28.9
	30.6 – 34.0	32.3	0	32.3
**TN 2014**				
	0.0 – 4.6	2.3	0	2.3
	4.6 – 9.2	6.9	1.2	5.6
	9.2 – 13.8	11.5	1.6	9.9
	13.8 – 18.4	16.1	15.3	0.8
	18.4 – 23.0	20.7	21.3	-0.6
	23.0 – 27.6	25.3	27.1	-1.8
	27.6 – 32.2	29.9	32.7	-2.8
	32.2 – 36.8	34.5	0	34.5
	36.8 – 41.4	39.1	0	39.1
	41.4 – 46.0	43.7	0	43.7
**SRP 2014**				
	0.0 – 0.3	0.15	0.4	-0.2
	0.3 – 0.6	0.45	1.3	-0.9
	0.6 – 0.9	0.75	0	0.8
	0.9 – 1.2	1.05	0	1.1
	1.2 – 1.5	1.35	0	1.4
	1.5 – 1.8	1.65	0	1.7

	1.8 – 2.1	1.95	0	2.0
	2.1 – 2.4	2.25	0	2.3
	2.4 – 2.7	2.55	0	2.6
	2.7 – 3.0	2.85	0	2.9
**TDP 2014**				
	0.0 – 0.2	0.1	0	0.1
	0.2 – 0.4	0.3	0.2	0.1
	0.4 – 0.6	0.5	0.8	-0.2
	0.6 – 0.8	0.7	2.0	-1.3
	0.8 – 1.0	0.9	5.2	-4.3
	1.0 – 1.2	1.1	9.9	-8.8
	1.2 – 1.4	1.3	0	1.3
	1.4 – 1.6	1.5	0	1.5
	1.6 – 1.8	1.7	0	1.7
	1.8 – 2.0	1.9	0	1.9
**TP 2014**				
	0 – 1	0.5	0.8	-0.3
	1 – 2	1.5	2.3	-0.9
	2 – 3	2.5	5.0	-2.5
	3 – 4	3.5	0	3.5
	4 – 5	4.5	0	4.5
	5 – 6	5.5	0	5.5
	6 – 7	6.5	0	6.5
	7 – 8	7.5	0	7.5
	8 – 9	8.5	0	8.5
	9 – 10	9.5	0	9.5
